# Mitomycins syntheses: a recent update

**DOI:** 10.3762/bjoc.5.33

**Published:** 2009-07-08

**Authors:** Jean-Christophe Andrez

**Affiliations:** 1 Department of Chemistry, University of British Columbia, 2036 Main Mall, Vancouver, BC, V6T1Z1, Canada

**Keywords:** antitumour, bioactivity, mitomycin, mitosene, synthesis

## Abstract

Mitomycins are a class of very potent antibacterial and anti-cancer compounds having a broad activity against a range of tumours. They have been used in clinics since the 1960’s, and the challenges represented by their total synthesis have challenged generations of chemists. Despite these chemical and medicinal features, these compounds, in racemic form, have succumbed to total synthesis only four times over the last 30 years.

## Review

### Introduction

The mitomycins pose unique challenges to the synthetic chemist. As S. Danishefsky noted, “The complexity of the problem arises from the need to accommodate highly interactive functionality in a rather compact matrix and to orchestrate the chemical progression such as to expose and maintain vulnerable structural elements as the synthesis unfolds. The synthesis of a mitomycin is the chemical equivalent of walking on egg shells.”

**Scheme 1:**
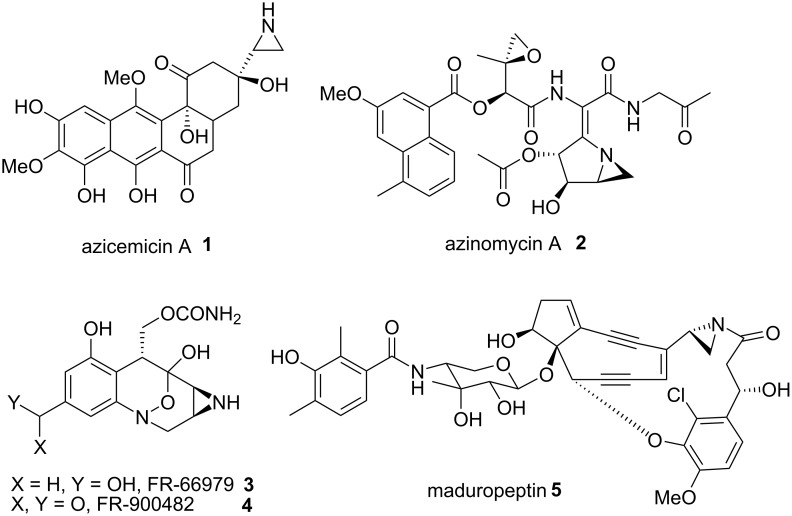
Aziridine containing natural products.

**Scheme 2:**
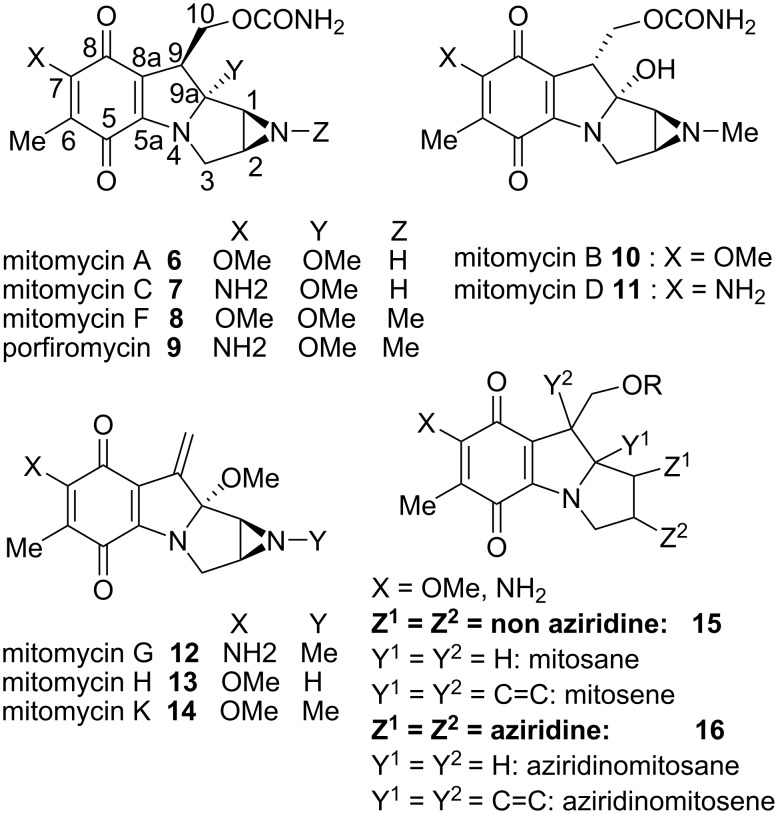
Mitomycin structures and nomenclature.

The first discovery of a mitomycin (mitomycin C, Scheme 2, compound **7**) dated from 1958 [1]. Its structural elucidation was remarkable at that time considering the presence of 4 contiguous stereogenic carbons in the molecule. The tetracyclic pyrrolo-indole skeleton of a mitomycin is embellished with an aziridine ring, a carbamoyl moiety and a bridged carbinolamine packed in a constrained architecture [2]. The presence of such a concentration of functional groups renders this molecule only moderately stable to bases, acid and nucleophiles but particularly reactive in presence of reducing agents. Notwithstanding their apparent fragility, mitomycins were rapidly identified to act as prodrugs and their unique activity was thought to originate from their ability to transform in vivo to generate the active metabolite. This was followed by decades of investigations to understand in detail their singular mode of action. It was found that the aziridine played a crucial role, allowing an irreversible bis-alkylation of DNA [3]. The decisive role of the aziridine is far from unusual since its presence in a small number of other naturally occurring molecules such as azinomycins [4–5], FR-900482 [6], maduropeptin [7], and azicemicins [8] is accompanied by significant biological properties (Scheme 1) [3,9]. Mitomycin C, **7**, the most potent mitomycin, has been used medicinally since the 1970’s for its activity against breast, stomach, oesophagus and bladder tumours [9]. Besides the well-known antibiotic and antitumour properties of these compounds [9–12], other semi-synthetic derivatives were prepared for investigation in clinical trials [13–14].

Altogether, the biological features of the mitomycins and the challenges represented by their total synthesis have continually drawn the attention of numerous brilliant chemists who conceived many different routes for their synthesis. However, only four total syntheses have been achieved [15–20]. Also, this review will summarize the current state of the art concerning the chemistry and biology of mitomycins. It will show and comment on the methodologies that have been successfully employed in total syntheses as well as approaches leading to mitomycin analogs. The review will focus on the synthetic literature of the past 30 years. From time to time earlier references will be provided to give background information. The mitomycin’s close cousins, the FR family (Scheme 1, compounds **3** and **4**) will not be discussed, nor will the different strategies that have been employed to improve the efficacy of mitomycins in vivo by structure activity relationship studies. As a matter of fact, the natural products themselves are so sensitive that only minor modifications have been possible in connection with medicinal chemistry studies.

## Discussion

### 1. Mitomycin isolation and nomenclature

Mitomycins are natural products isolated from extracts of genus *Streptomyces*, a filamentous gram-positive soil bacterium that produces a wide array of biologically active compounds, including over two-thirds of the commercially important natural-product metabolites [21]. Mitomycin C is extracted from the bacterium *Streptomyces lavendulae* and is far from the most known compound of the series. It has become one of the most effective drugs against non-small-cell lung carcinoma, as well as other soft and solid tumours [22]. The seven most abundant mitomycins (A to K) in nature are presented in Scheme 2.

Since many synthetic attempts did not succeed in providing mitomycins per se but only close relatives of these molecules, a special nomenclature has been elaborated for these compounds: structures of type **15**, which do not contain an aziridine ring, but bear the *p*-quinone are called mitosanes or mitosenes depending whether they are at the oxidation state of an indoline (Y^1^ or Y^2^ = H) or an indole (Y^1^ = Y^2^ = C=C). Compounds bearing an aziridine at C1 and C2 are described specifically as aziridinomitosanes and aziridinomitosenes respectively. Compounds possessing a hydroquinone (protected or not) in place of the original quinone are identified by inclusion of the prefix *leuco* in reference to the Greek word *leukos* (clear, white) and the lack of intense color usually specific of the corresponding quinone ring.

**Scheme 3:**
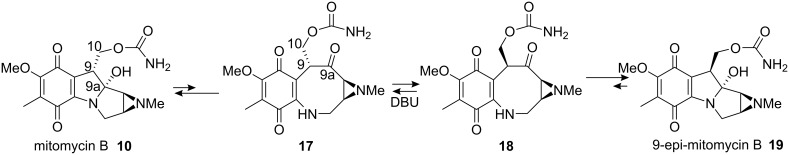
Base catalysed epimerization of mitomycin B.

The mitomycins A and C differ only by the substituents on the quinone ring and transformation from **6** to **7** is realized by simple treatment with ammonia [23–24]. The mitomycins F, **8**, and porfiromycin, **9**, are synthesized by methylation of the aziridine of mitomycin A and mitomycin C, respectively. Mitomycin G, **12**, mitomycin H, **13**, and mitomycin K, **14**, are derivatives of this first series obtained by elimination of the carbamate at position 10 [25–26]. Mitomycin B, **10**, and mitomycin D, **11**, possess the opposite absolute configuration of the asymmetric carbon C9. Interestingly, Hornemann proved that this carbon could be easily epimerized to give 9-epi-mitomycin B, **19**. This compound showed better activity than the non-epimerized one. He proposed a based-catalyzed mechanism wherein the tetracyclic-pyrolido-indole structure **10** opens up reversibly at the bridged carbinolamine junction to give the eight-membered ring **17**. The base, in this case DBU, then reversibly deprotonates the activated C9 position to give the more stable isomer **18** (Scheme 3) [27]. The basis of this surmise was the finding that mitomycin B eliminates carbamic acid at the C10 position in basic medium whereas the angular methoxy series (mitomycin C), which can not open to the amino ketone, needs a better leaving group (such as a sulfonate).

### 2. Biology

#### 2.1. Biosynthesis

**Scheme 4:**
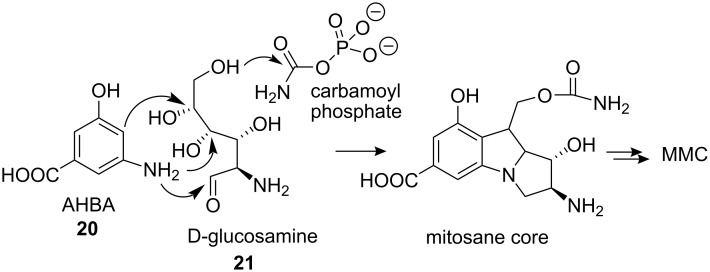
Biosynthesis of mitomycin C (MMC) **7**.

A significant amount of information on the biosynthesis of mitomycin C has been accumulated since 1970 [28]. The mitosane core was shown to be derived from combination of 3-amino-5-hydroxybenzoic acid **20** (AHBA), D-glucosamine **21** and carbamoyl phosphate (Scheme 4) [29–32]. The key intermediate, AHBA, is also a common precursor to other anticancer drugs, such as rifamycin and ansamycin.

#### 2.2. Mode of action

**Scheme 5:**
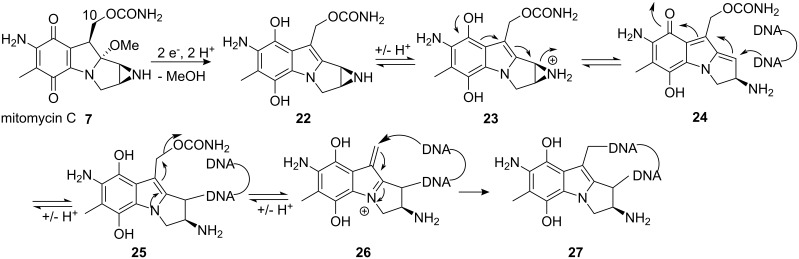
Mode of action of mitomycin C.

Mitomycins are quinone antitumor antibiotics that exert their biological activity through DNA alkylation and cross-linking. The success of mitomycin C in cancer treatment is due to a great cytotoxic selectivity for hypoxic (O_2_-deficient) cells characteristic of solid tumors [33–34]. Mitomycin C itself is indeed relatively unreactive toward DNA [35–36] but becomes remarkably reactive upon reduction (enzymatic, electrochemical or chemical) by the mechanism shown in Scheme 5 [37–39]. This mechanism was proposed 40 years ago based purely on structural considerations and has been accepted since [40]. Only the first reductive activation step has been questioned as to whether it proceeds by a one-electron reduction to give the semiquinone [41–44] or by a two-electron reduction to give the hydroquinone [45–47]. Studies have shown that one-electron reduction in an organic solvent can trigger formation of the semiquinone and the subsequent reaction cascade [42]. On the other hand, two-electron reduction led to formation of the stable hydroquinone, which can be oxidized back to the quinone in the presence of oxygen [48]. Nonetheless, different results were observed in water where both one- and two-electron reductions gave the same DNA adducts. Moreover, the disproportion of the semiquinone in aqueous anaerobic medium is also very fast [49] whereas under aqueous aerobic conditions, the semiquinone reoxidizes to the quinone more quickly than it disproportionates [50]. Thus, the conclusion was made that in aqueous medium the same hydroquinone intermediate was responsible for the reaction cascade.

The following section will focus on different synthetic approaches to mitomycins and will be divided into seven sections, each corresponding to a retrosynthetic disconnection involving at least one common bond formation. Part 9 will deal with *miscellaneous* disconnections.

### 3. The N–C3–C9a disconnection

**Scheme 6:**
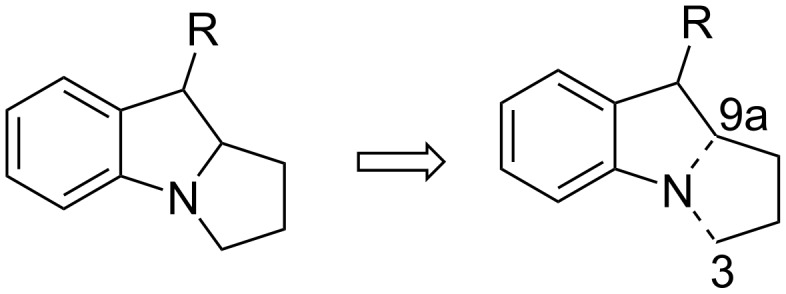
The N–C3–C9a disconnection.

#### 3.1. Danishefsky. Mitomycin K (MMK)

**Scheme 7:**
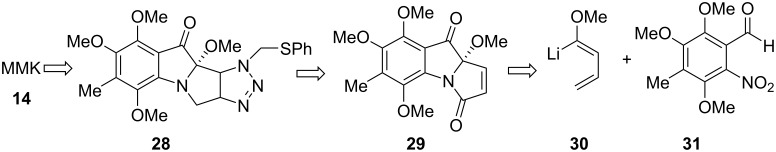
Danishefsky’s Retrosynthesis of mitomycin K.

The retrosynthetic approach of Danishefsky is based on an intramolecular Diels–Alder reaction between a nitrosoaryl and a suitably functionalized diene (Scheme 7) [19]. Historically, this strategy was designed to synthesize the related natural product FR-900482, **4**, but their investigations also led to this significant and concise total synthesis of mitomycin K. In fact, assembly of the tetracyclic structure occured in only four steps, with all the key structures in place, making their synthesis very practical. Another key reaction was the efficient introduction of the *N*-methyl aziridine in only three steps from an olefin by cycloaddition of methylthiophenyl azide onto the unsaturated amide **29**.

**Scheme 8:**
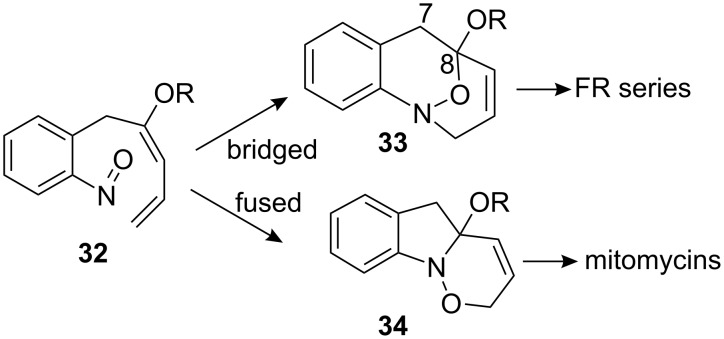
Hetero Diels–Alder reaction en *route* to mitomycins.

As mentioned before, the construction of FR-900482 was thought to occur by intramolecular hetero Diels–Alder reaction of a compound of type **32** (Scheme 8) [51]. After careful analysis, it was envisioned that the reaction could occur either in the bridged mode to give the FR series (compounds **3** and **4**) or in the fused mode to give access to mitomycins. Further investigations confirmed that the intramolecular hetero Diels–Alder reaction favoured the fused mode and did not constitute a viable route for the synthesis of the FR series. Nonetheless, changing the approach by using an intermolecular Diels–Alder reaction gave efficiently the bridged adduct and allowed one of the most elegant total syntheses of FR-900482 [52]. As a result, this astonishing synthesis of mitomycin K can be seen as a “by-product” of the synthesis of FR-900482.

**Scheme 9:**
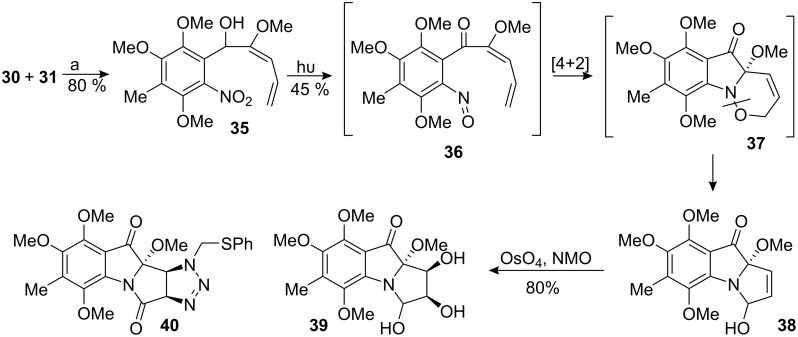
Nitroso Diels–Alder cycloaddition.

The functionalized nitroso-diene **36** was efficiently formed by photochemical rearrangement of the nitro-carbinol **35**, which arose from the addition of vinyl lithium **30** into aldehyde **31** (Scheme 9). Further irradiation at 350 nm triggered a cycloaddition to give compound **38**, probably through intermediate **37**. Deserving of mention is the unusual instability of compound **37** under the reaction conditions, which can be attributed to the high captodative stabilization of the aniline radical formed upon cleavage of the nitrogen-oxygen bond. Functionalization of the olefin in compound **38** was accomplished first by dihydroxylation with osmium tetroxide. The reaction was stereospecific, resulting in formation of the diol **39** derived from attack of the reagent from the concave face of the molecule.

**Scheme 10:**
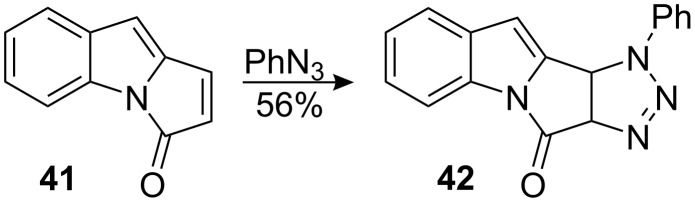
Frank azide cycloadddition.

Diol **39** was found to have the undesired stereochemistry for the planned construction of the aziridine by tandem S_N_2 displacement. Exploiting the facial selectivity of compound **38**, the direct introduction of an aziridine equivalent was attempted by 1,3-dipolar cycloaddition of an alkyl azide. As Frank noted, cycloaddition of azides to 3*H*-pyrrolo [1,2-a]indoles gives rise to complex reaction mixtures due to the possibility of nitrene insertion. Cycloaddition of phenyl azide, however, to the unsaturated carbonyl **41** was readily accomplished to give triazoline **42** in 56% yield (Scheme 10) [53–55].

**Scheme 11:**
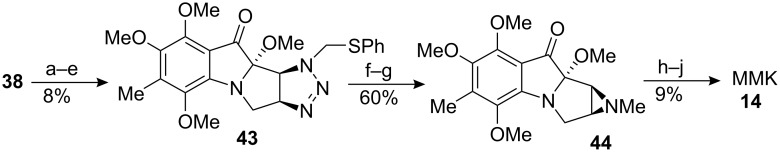
Final steps of mitomycin K synthesis. ^a^PDC, DCM; ^b^PhSCH_2_N_3_, PhH, 80 °C; ^c^L-selectride, THF, −78 °C; ^d^1,1′-(thiocarbonyl)diimidazole, DMAP, DCM, 35 °C; ^e^Bu_3_SnH, AIBN, PhH, 80 °C; ^f^hυ 254nm, PhH; ^g^RaNi, AcOH, 60 °C; ^h^[(trimethylsilyl)methyl]lithium, THF, −10 °C; ^i^silver (II) pinacolate, NaOAc, CH_3_CN/H_2_O; ^j^PPTS, DCM.

According to this scheme, the allylic alcohol **38** was oxidized with pyridinium dichromate and reacted with methylthiophenyl azide [56] to give the triazoline **40** derived from attack of the reagent from the concave face of the molecule with high diastereoselectivity (Scheme 11). The electronic effect or the α-methoxy group, as well as shielding of the α-face of the molecule, provides the desired steroselectivity.

An uncommon transformation was then accomplished: the reduction, with L-Selectride, of a lactam in the presence of a ketone. There are several causes for the unusual chemoselectivity of this reduction. The lone pair electrons of the amide nitrogen in compound **40** are conjugated with the ketone through the aromatic ring. Thus, the lactam behaves as an imide whose carbonyls express ketone-like reactivity. Moreover, the upper carbonyl is deactivated by conjugation with a methoxy group in the ortho position of the benzene ring, and by the steric crowding by both this methoxy group and the one at the ring junction. Finally, the nitrogen of the lactam is located in the bridgehead position of a [3.3.0] bicycle resulting in poor delocalization into the adjacent carbonyl.

A Barton deoxygenation gave compound **43**, which was irradiated to decompose the triazoline to an aziridine. The thiophenyl component was then removed with Raney nickel, which provided an elegant way to introduce the N-methyl group of the aziridine. Compound **44** was then treated with (trimethylsilyl)methyl lithium to install the exocyclic olefin via Peterson’s method. The *p*-dimethoxyhydroquinone was then oxidized with silver(II) pinacolate (in poor yield) to give mitomycin K.

#### 3.2. Naruta, Maruyama. Azide cycloaddition

**Scheme 12:**
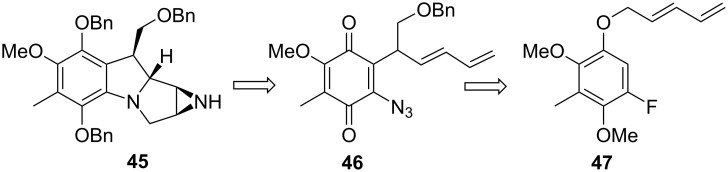
Naruta–Maruyama retrosynthesis.

From a synthetic point of view, the intramolecular [1+4] cyclisation of a nitrene with a dienyl moiety gives a pyrrolizidine, the key structure found in mitomycins and many other mammal and vegetal alkaloids. The Naruta–Maruyama group exploited this reaction for the synthesis of a leucoaziridinomitosane based on the retrosynthesis shown in Scheme 12 [57–58].

**Scheme 13:**
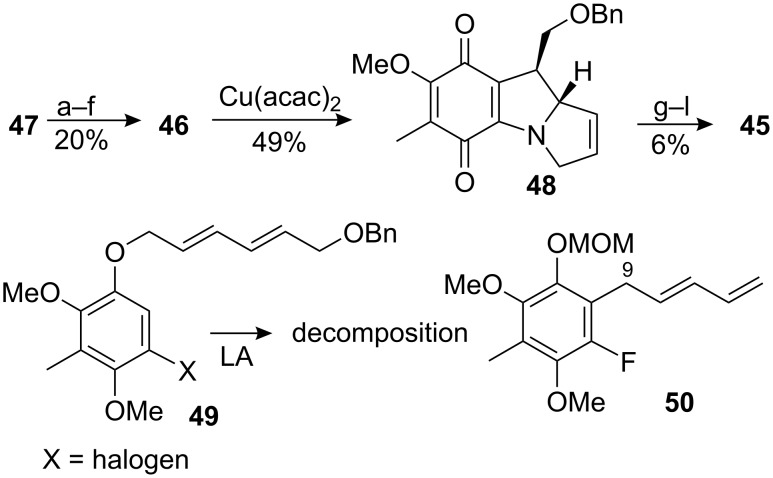
Synthesis of a leucoaziridinomitosane by nitrene cycloaddition. ^a^AlCl_3_-Et_2_O; ^b^NaH, ClCH_2_OMe; ^c^*n*-BuLi, ClCH_2_OBn; ^d^H_3_O^+^; ^e^CAN; ^f^NaN_3_; ^g^Zn, AcOH; ^h^BnBr, K_2_CO_3_; ^i^OsO_4_, NMO; ^j^MsCl, Et_3_N; ^k^*n*-Bu_4_N_3_; ^l^MsCl, Et_3_N; ^m^P(OMe)_3_; ^n^NaH; ^o^LiAlH_4_.

The synthesis began with a Claisen rearrangement of the pentadienyl aryl ether **47** under Lewis acidic conditions (Scheme 13). After protection of the resulting phenol with a MOM group, the regioselective introduction of an alkoxymethyl group at the C9 position, the most crowded location on the pentadienyl moiety of compound **50**, was the next obstacle. The best results were obtained by forming the pentadienyl anion with butyllithium followed by quenching with benzyloxymethyl chloride. The desired compound was obtained in 55% yield while the other regioisomers were isolated in a combined 22% yield. The more direct approach using the alkoxymethyl substituted pentadienyl aryl ether **49** failed to give the Claisen rearrangement product.

**Scheme 14:**
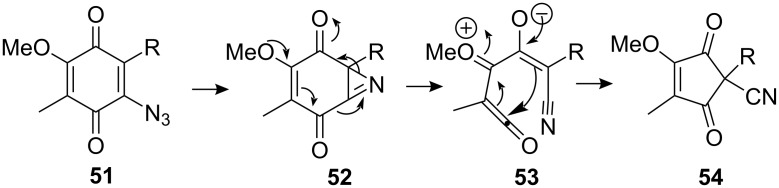
Thermal decomposition of azidoquinone **51**.

The MOM group was then removed, the phenol oxidized to the *p*-quinone with CAN and the fluorine displaced by azide to give compound **46**. Decomposition of azide-containing olefins have been performed under thermal, photolytic, acid-catalyzed or transition metal-catalyzed conditions [59]. With this substrate the subsequent cyclisation of azidodienylquinone **46** was performed with Cu(acac)_2_ as catalyst and afforded **48** with a high degree of diastereoselectivity. Thermal reaction led to the formation of the ring-contracted cyclopentendione derivative **54**. The reaction was thought to proceed by attack of the quinone by an intermediate nitrene via intermediates **52** and **53** (Scheme 14) [60].

**Scheme 15:**
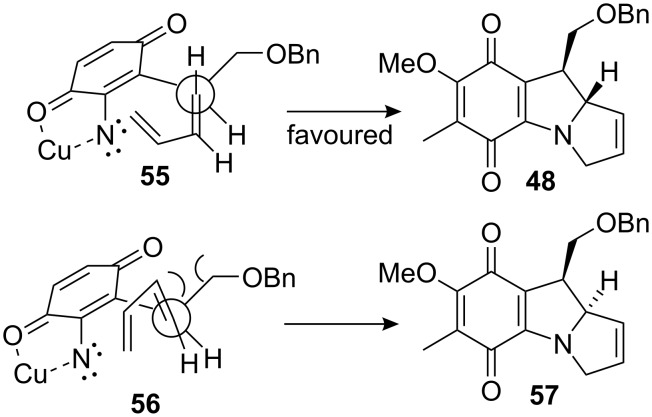
Diastereoselectivity during the cycloaddition.

The diastereoselectivity observed during the cycloaddition originated from the most favored staggered conformation in the transition state based on the Houk model, wherein the allylic hydrogen is eclipsed by the olefinic hydrogen (compound **55**) to minimize 1,3-allylic strain (Scheme 15) [61].

**Scheme 16:**
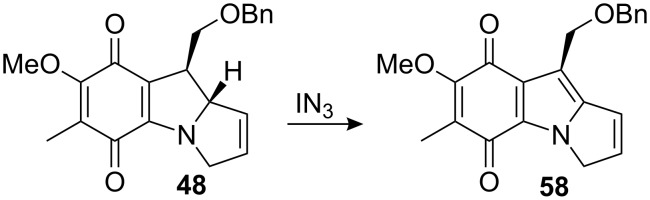
Oxidation with iodo-azide.

Attempts to introduce the aziridine from the olefin **48** using halogeno-azides like BrN_3_ or IN_3_ and subsequent reduction failed because the compound oxidized rapidly to the indoloquinone **58** (Scheme 16).

A longer sequence using dihydroxylation with osmium tetroxide, mesylation and displacement with azide was used to produce leucoaziridinomitosane **45**, whose spectral data matched those of an authentic sample derived from natural mitomycin C.

#### 3.3. Williams. Mitsunobu reaction

**Scheme 17:**
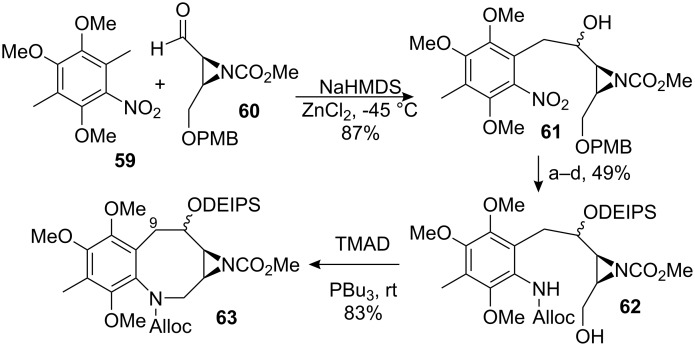
Williams’ approach towards mitomycins.^a^DEIPSCl, Imidazole, DCM; ^b^Pd/C, HCO_2_NH_4_, MeOH; ^c^AllocCl, NaHCO_3_, DCM-H_2_O; ^d^DDQ, CH_2_Cl_2_.

R.M. Williams successfully used this disconnection during the total synthesis of FR-900482 [62–63]. Coupling of nitrotoluene **59** and aldehyde **60** [64] gave the aldol product **61** as a 2:1 mixture of diastereomers (Scheme 17). Manipulation of protecting groups and oxidation states led to compound **62**, which cyclised smoothly under Mitsunobu conditions to form the eight-membered ring of **63** [65]. Unfortunately, all attempts to introduce the hydroxymethyl group at C9 by aldol-type strategies met with failure. Electronics seem to play a major role, as very similar substrates *en route* to FR-900482 underwent analogous reactions [66]. Solving this issue at the stage of the benzazocenol or at an earlier intermediate would constitute a major breakthrough.

#### 3.4. Danishefsky. Homoconjugate addition

**Scheme 18:**
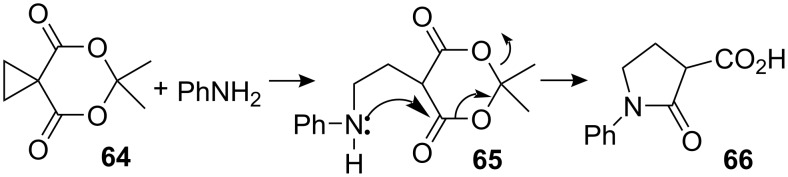
Synthesis of pyrrolidones by homoconjugate addition.

The homoconjugate opening of activated cyclopropanes has been studied by many groups, utilizing all kinds of nucleophiles [67–70]. Although this methodology was known before 1900 [71], it did not draw much attention in total synthesis because of its lack of efficiency. In the middle of 1970’s Danishefsky and co-workers focused on the systematic study of this reaction and made relevant improvements for both the preparation of the cyclopropane and the enhancement of reactivity of the cyclic acylal **64** [72–76]. This methodology has seen application in the synthesis of diverse heterocycles, as exemplified by the reaction of aniline with **64** to form the 1,5-addition adduct **65**, and finally the amide **66** by internal acylation and extrusion of acetone (Scheme 18).

**Scheme 19:**
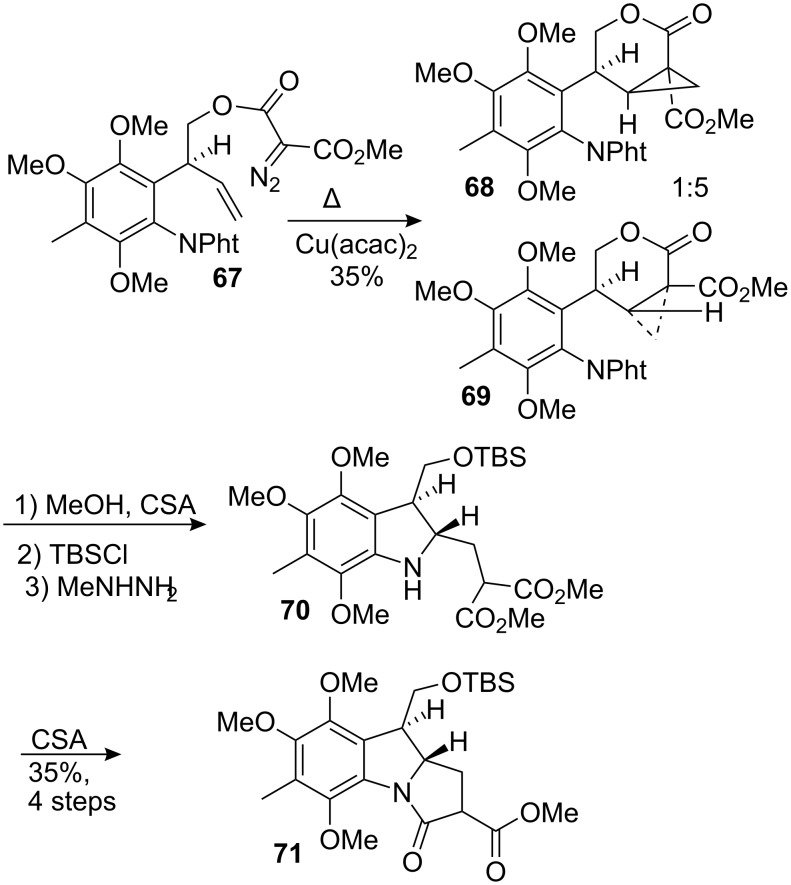
Homoconjugate addition on the fully functionalized substrate.

This methodology provided a new entry for the construction of pyrrolidine-indole structures based on the intramolecular opening of an activated cyclopropane [75–77]. This work culminated in the synthesis of an advanced intermediate en route to the mitosane series [78]. Compound **67** was thermolyzed in refluxing chlorobenzene in the presence of cupric acetonylacetonate to give the cyclopropane adduct in 35% yield (Scheme 19) [79]. The product arose as a 5:1 mixture of diastereoisomers, with the major component **69** having the bulky hexasubstituted phenyl group on the convex face of the bicyclic[4.1.0] ring system. It was necessary to methanolyse the lactone **69** prior to removal of the phthalimide with methyl hydrazine, which occurred with concomitant cyclization to form penultimate intermediate **70**. The final ring closure was realized by treatment with camphorsulfonic acid to form lactam **71** in 35% yield over 4 steps.

**Scheme 20:**
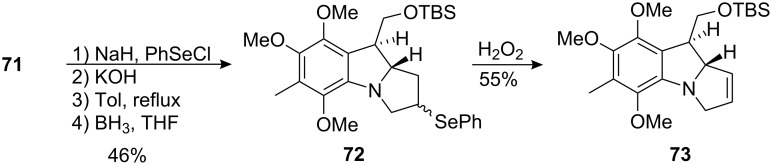
Introduction of the olefin.

The transformation of this compound into an intermediate suitable for the introduction of an aziridine was found to be extremely difficult. A sequence leading to the formation of the olefin **73** was enacted. This compound was thought to be a good candidate for the installation of the aziridine. They exploited the presence of a β-dicarbonyl functionality in **71** to introduce a phenylseleno function α to the lactam centre (Scheme 20). Then, hydrolysis of the methyl ester followed by decarboxylation and reduction of the lactam with borane gave the selenide diastereoisomers **72** in 46% overall yield. Interestingly, oxidation with hydrogen peroxide gave predominantly the N-allylic system rather than the vinylic one. This is in accordance with the precedent established by the laboratory of K.B. Sharpless [80–81].

The overall yield from diazo-ester **67** was only 2% and was deemed insufficient to pursue further synthetic study. However, the idea of a tandem reaction opened the door to more innovative and fascinating syntheses.

#### 3.5. Danishefsky. One pot N–C9a, N–C3 formation

**Scheme 21:**
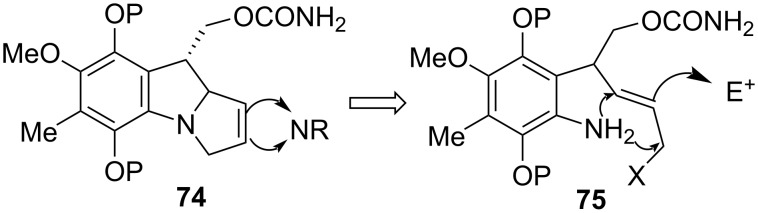
Retrosynthesis of N–C9a, N–C3 bond formation.

In the early 1980’s the Danishefsky group envisioned the possibility of the diastereoselective introduction of the aziridine via the olefinic portion of a pyrroline such as **74** [82]. The construction of this compound would proceed through a sequence such as that depicted in Scheme 21. Upon electrophilic activation of the olefin **75**, the aniline would attack it to form a pyrroline ring, making the terminal leaving group X free for nucleophilic displacement with the nitrogen of the newly formed pyrroline.

**Scheme 22:**
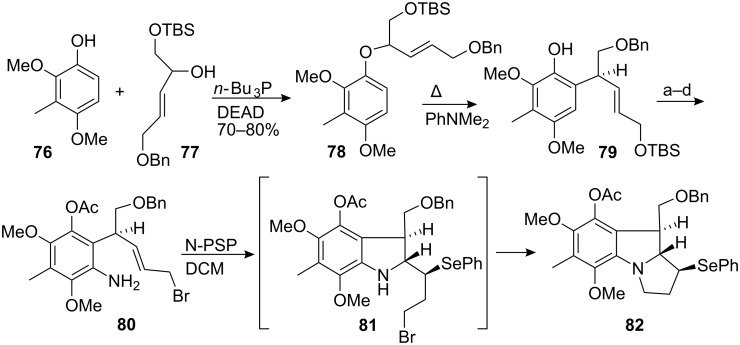
Synthesis of the pyrrolo[1,2]indole **82** using N-PSP activation.^a^Ac_2_O, Py; ^b^Ac_2_O, Hg(OAc)_2_, AcOH, 90% HNO_3_; ^c^Zn, HCl, MeOH; ^d^CBr_4_, PPh_3_, Et_2_O. (31% overall yield from **78**).

The synthesis began by Mitsunobu coupling of phenol **76** with allylic alcohol **77** to give ether **78**, which was heated in N,N-dimethylaniline to trigger a Claisen rearrangement (Scheme 22). A sequence of straightforward reactions then led to compound **80**. As expected, the *trans* double bond in **80** prevented intramolecular alkylation of the amino group by the homoallylic bromide, thus explaining the somewhat surprising stability of this compound. The key step cyclisation was then carried out with the Nicolaou’s reagent, N-phenylselenophthalimide (N-PSP) [83–84]. The attack of this reagent upon the double bond led to indoline **81** which underwent a second alkylation to generate the complete pyrroloindole system **82**.

It is noteworthy that the cyclization reaction was completely stereospecific. Indeed, one could argue that the addition of N-PSP to the olefin **80** to form a selenonium ion is reversible and that the attack of the nitrogen is favored when the two large groups emerge *trans* relative to the indoline ring.

**Scheme 23:**
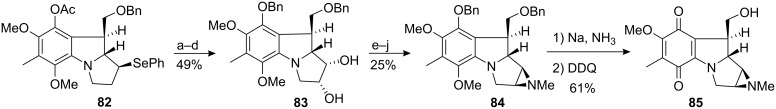
Synthesis of an aziridinomitosane. ^a^*m*-CPBA, DCM then iPr_2_NH, CCl_4_ reflux; ^b^K_2_CO_3_, MeOH; ^c^BnBr, KH; ^d^OsO_4_, NMO; ^e^MsCl, Et_3_N; ^f^*n*-Bu_4_NN_3_, C_6_H_6_; ^g^MsCl, Et_3_N; ^h^P(OMe)_3_ then NaH; ^i^LAH; ^j^MeLi, MeI.

Treatment with *m*-CPBA then created the double bond which was later fashioned into aziridine **84** (Scheme 23). First, the phenol acetate was replaced by a benzyl group. Among the different oxidants screened for dihydroxylation, osmium tetroxide was preferred, since it did not effect aromatization of the indoline ring and gave diol **83** as a single isomer. Dihydroxylation occurred selectively from the concave face of the molecule, *anti* to the *exo*-disposed benzyloxymethyl group. The standard methodology involving azide displacement gave the aziridine **84**. The benzyl groups were removed using a Birch reduction and subsequent oxidation with DDQ furnished the aziridinomitosane **85** in good overall yield.

**Scheme 24:**
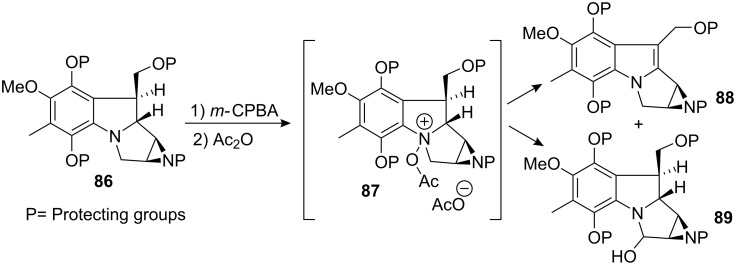
Oxidation products of a leucoaziridinomitosane obtained from a Polonovski oxidation.

Although this scheme was suitable for the synthesis of aziridinomitosanes, a venture to introduce the C9a heteroatom functionality (essential for activity [85]) was attempted unsuccessfully in this study. The same authors observed that any attempts to oxidize a leucoaziridinomitosane of type **86** via a Polonovski reaction inevitably gave either the corresponding leucoaziridinomitosene **88** or the oxidation product at C3, **89** (Scheme 24) [86]. The N-oxo-acetyl intermediate **87** can eliminate an acetate group upon deprotonation at carbon C9a or C3 leading to two regioisomeric iminium species. The iminium formed by deprotonation at carbon C9a probably rearranged to leucoaziridinomitosene **88** faster than it was trapped by a nucleophile. The putative attack of an nucleophile on the transient iminium species would create an equilibrium with the starting iminium which would eventually convert to the thermodynamically more stable indole **88**.

**Scheme 25:**
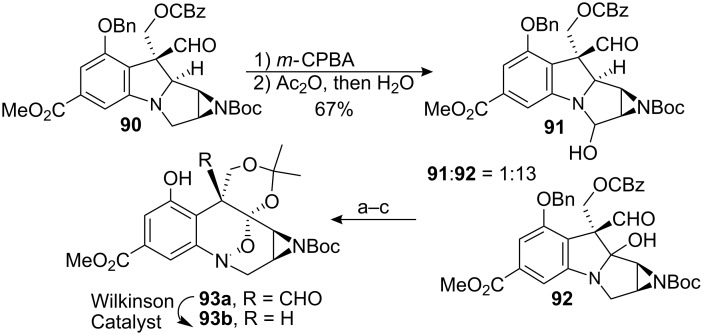
Polonovski oxidation of an aziridinomitosane. ^a^*m*-CPBA; ^b^Pd/C, H_2_; ^c^Dimethoxypropane, PPTS.

More recently, a suitable substrate for the selective oxygenation of the C9a position of a mitomycin derivative was discovered by F.E. Ziegler and co-workers [87]. The use of a Polonovski reaction [88] on the aziridinomitosane **90** gave the C9a oxygenated compound **92** in 67% yield (Scheme 25). Rewardingly, the selectivity for this reaction was good, giving only minor quantities of the C3 oxidation product **91**.

A possible explanation for this selectivity is the participation of the carbonyl of the carbobenzyloxy protecting group in the elimination process by internally assisting deprotonation at C9a through a seven-membered ring and thereby accelerating the formation of the desired iminium ion. The authors found that changing the carbobenzyloxy protecting group to less basic groups such as a silyloxy ether or a methoxymethyl ether gave virtually no selectivity between C9a and C3 oxidation. In an attempt to conclude the synthesis of FR-900482, substrate **93a** was subjected to decarbonylation conditions, using 2.2 equivalents of Wilkinson’s catalyst, (PPh_3_)_3_RhCl, to give the corresponding decarbonylated product **93b** with retention of configuration of the C10 side chain. However, the reaction proved to be capricious and inconsistent results were routinely observed making the synthesis not very practical.

### 4. The C1–C9a disconnection

**Scheme 26:**
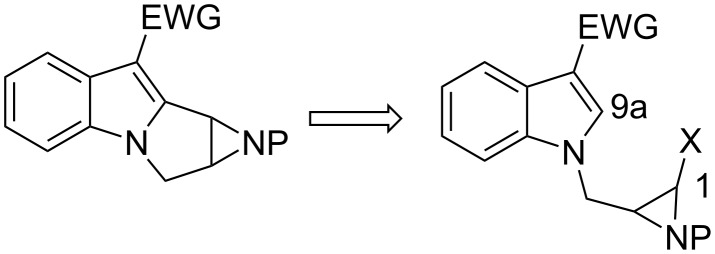
The C1–C9a disconnection.

#### 4.1. Ziegler. Aziridinyl radical

**Scheme 27:**
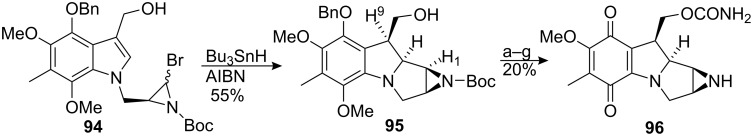
Ziegler synthesis of desmethoxymitomycin A.^a^Im_2_C=O, THF; ^b^NH_3_; ^c^TMSOTf, 2,6-di-*tert*-butylpyridine, DCM; ^d^Ac_2_O, Et_3_N; ^e^H_2_, Pd/C, EtOAc; ^f^DDQ, −78 °C→25 °C; ^g^NH_3_, MeOH.

**Scheme 28:**
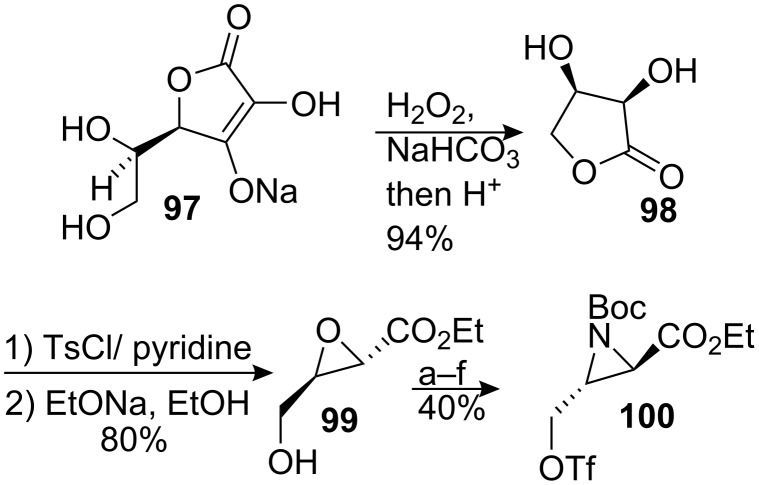
Transformation of sodium erythorbate.^a^TBDMSCl; ^b^NaN_3_; ^c^PPh_3_; ^d^(Boc)_2_O, DMAP; ^e^TBAF; ^f^Tf_2_O, Pyr.

Ziegler and Berlin proposed a disconnection for the synthesis of aziridinomitosanes based on a 5-exo-trig cyclisation of an aziridinyl radical (Scheme 27) [89]. This approach allowed stereocontrolled, rapid access to either enantiomer of this type of structure. Compound **94** was obtained by S_N_2 displacement with chiral aziridinyl triflate **100** followed by decarboxylation. Among the numerous routes to synthesize compound **100** [90–92] the authors opted for an economical way starting from the common food preservative sodium erythorbate **97**, the enantiomer of the sodium salt of ascorbic acid (vitamin C) (Scheme 28). It was transformed in three steps into the (2*S*,3*R*)-4-hydroxy-2,3-epoxybutyrate **99** [93] which was in turn advanced to the triflate aziridine **100** by the general procedure developed by Blum [94]. The cyclisation compound **94** took place by generating the aziridinyl radical in the presence of tributyltin hydride and a radical initiator, azobisisobutyronitrile, in refluxing toluene. The transient radical that formed at C9 then abstracted a hydrogen atom from *n*-Bu_3_SnH to the convex face of the molecule, giving leucoaziridinomitosane **95** with a *cis* relationship between H9 and H1. The desmethoxymitomycin A **96** was then elaborated using standard protocols.

**Scheme 29:**
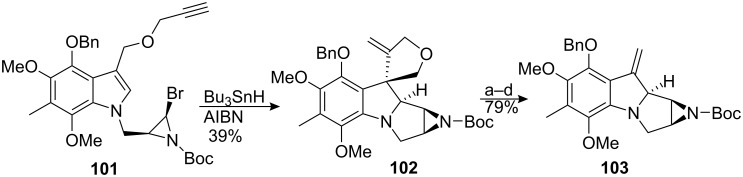
Formation of C9,C10-unsaturation in the mitomycins. ^a^*m*-CPBA, DCM; ^b^O_3_, MeOH; ^c^Me_2_S; ^d^KHMDS, (EtO)_3_P, O_2_, THF.

The oxidative introduction of a methoxy group at the C9a position remains challenging. Recently, the Ziegler group disclosed an interesting opportunity to achieve this goal in a related study (Scheme 29) [95]. Keeping in mind that oxidation of the C9a position would be possible if the C9 is quaternized, this group focused on the idea of introducing a protecting group at C9 which, upon liberation, would reveal the C9–C10 double bond found in mitomycin K. Applying the same radical process depicted in Scheme 27 with substrate **101**, a tandem cyclization was observed to afford pentacycle **102**. The basic pyrrolidine nitrogen was protected as its N-oxide and the exocyclic olefin was then converted to the corresponding ketone through ozonolysis. The ozonide and N-oxide were reduced with dimethyl sulfide at the end of the reaction. Using the Gardner protocol [96] to hydroxylate the alpha position of the resultant ketone failed to give any α-ketol, but instead provided directly the desired styrene **103** in good overall yield.

**Scheme 30:**
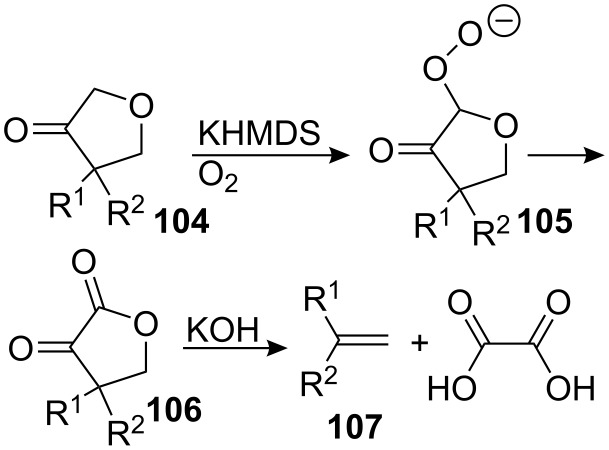
Fragmentation mechanism.

A possible explanation for this fragmentation is outlined in Scheme 30. The enolate of ketone **104** reacts with oxygen to form the peroxide anion **105**, which cleaves to give the α-keto-γ−butyrolactone **106**. Treatment of the latter with aqueous base generates the styrene **107** and oxalic acid.

#### 4.2. Vedejs. Anionic Michael addition

This strategy is very similar to Ziegler’s preceding approach since both share the same method for forming the C3–N4 bond and aim at constructing the C1–C9a bond. But while Ziegler uses a tandem radical cyclization, Vedejs employs an anionic Michael addition to form the C1–C9a bond [97].

**Scheme 31:**
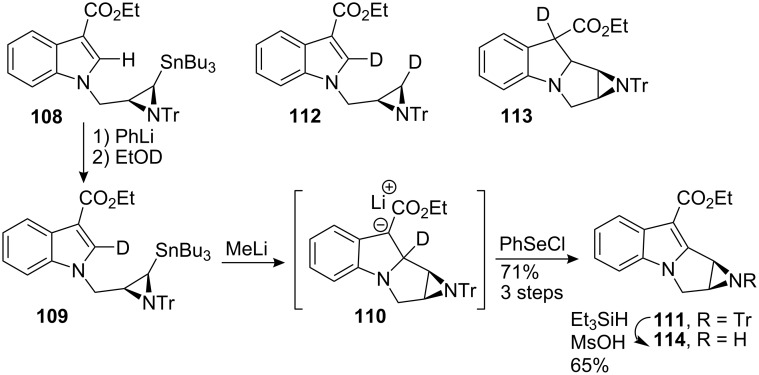
Michael addition-cyclisation.

Interestingly, preliminary attempts to effect metal exchange and internal Michael addition revealed a complex situation. Treating stannane **108** with an excess of methyl lithium followed by quenching with deuterated ethanol provided the monodeuterio derivative **109**, the corresponding de-stannylated dideuterio structure **112** and a small amount of the desired tetracyclic **113** (**109**:**112**:**113** = 55:36:9). This suggested that deprotonation of the indole was faster than the lithium metal exchange and prevented the desired Michael addition. They rationalized that monodeuterio derivative **109** should be a better substrate, having a slower indole deprotonation rate, due to a primary kinetic isotope effect. Accordingly, treatment of the monodeuterio derivative **109** with methyl lithium followed by quenching with phenylselenium chloride yielded directly the mitosene **111** (Scheme 31). As expected, in this case, the lithium-metal exchange occurred much faster and inverted the ratio of tetracyclic:tricyclic product from 1:10 to 4:1.

Removal of the trityl protecting group was then achieved using triethylsilane and methanesulfonic acid to give the parent free aziridine **114** in 65% yield. Although these types of aziridinomitosene are usually very unstable and aziridine solvolysis products are often formed, the presence of a deactivating ester group promoted the stability of the molecule.

#### 4.3. Reissig. Addition of samarium ketyls to alkynes

**Scheme 32:**
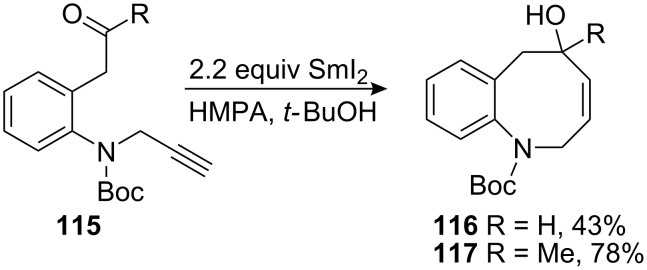
SmI_2_ 8-*endo*-*dig* cyclisation.

The direct synthesis of eight membered rings such as benzazocenol **116** is highly desirable since it provides a straightforward synthesis of mitomycins. However, the formation of these ring sizes from acyclic precursors is entropically and enthalpically disfavoured. Therefore, their synthesis became a great challenge in recent years [98]. In this regard, Reissig recently developed an 8-*endo*-*dig* cyclisation to give benzazocenols **116** and **117** using samarium iodide in moderate yields (Scheme 32) [99].

In these studies, simplified model compounds unsuitable for the synthesis of mitomycins were used and no further developments have been reported on this series of compounds.

#### 4.4. Jones. Radical cyclization

**Scheme 33:**
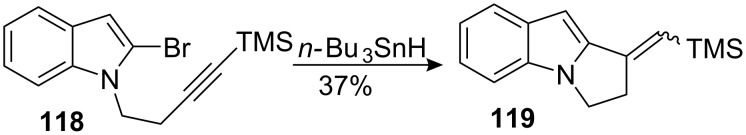
Synthesis of pyrrolo[1,2-a]indole by 5-*exo*-*dig* radical cyclization.

A radical cyclization was used by the Jones group for the formation of smaller rings via a 5-*exo*-*dig* radical cyclization. Bromoalkyne **118** was chosen to construct pyrrolo[1,2-a]indole **119**. The reaction was initiated with tributyltin hydride and gave the cyclized product **119** in 37% yield. (Scheme 33) [100].

Although compound **119** was not used in further studies, it might serve as a good forerunner for the synthesis of mitosenes as shown by Remers and discussed in section 6.3 of this review.

### 5. The C9–C9a disconnection

**Scheme 34:**
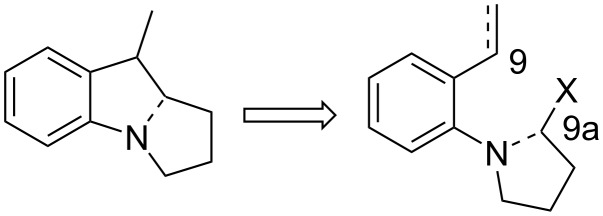
The C9–C9a disconnection.

#### 5.1. Kozikowski. Nitrile oxide cycloaddition, INOC

**Scheme 35:**
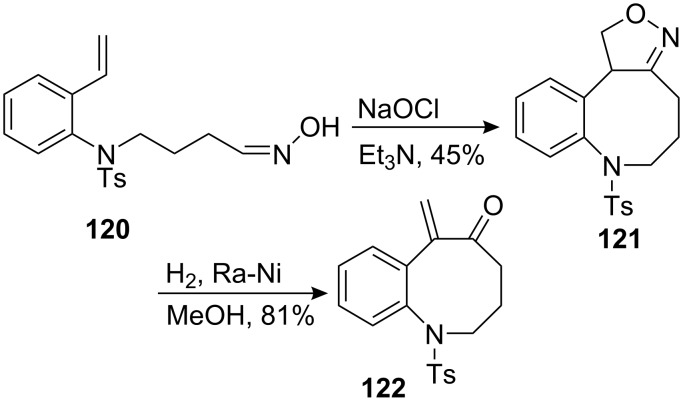
Intramolecular nitrile oxide cycloaddition.

The Kozikowski group used an intramolecular nitrile oxide cycloaddition (INOC) to form the eight membered ring of a benzazocine system [101]. The hydroxylamine **120** was treated with sodium hypochlorite to generate in situ a nitrile oxide which reacted with the terminal olefin to form the eight membered ring **121** (Scheme 35). Easy cleavage of the nitrogen–oxygen bond was realized using Raney nickel and the unsaturated ketone **122** was obtained in good yield.

**Scheme 36:**
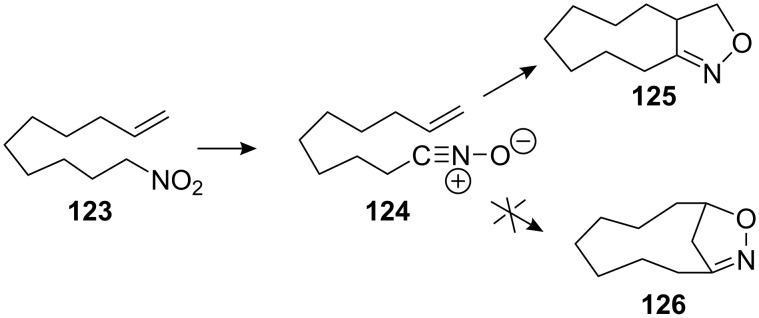
Regioselectivity of the INOC.

It is known that when a medium-sized ring is being generated by the INOC process none of the “normal” 5-substituted isoxazoline is formed. Hence, the nitrodecene **123** gives rise to only the nine-membered carbocycle **125** upon reaction with phenyl isocyanate. The matching HOMO-LUMO interactions for such cycloadditions favour the formation of **126** but ring strain and transannular steric effects oppose this orientation (Scheme 36).

**Scheme 37:**
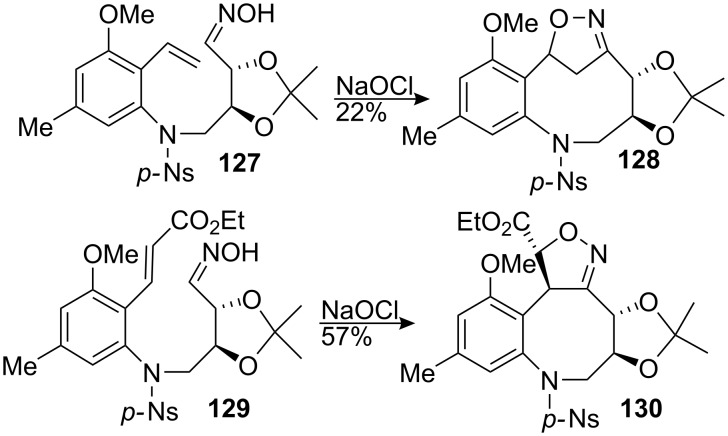
Fukuyama’s INOC strategy.

This methodology was used later on by the Fukuyama group to achieve the synthesis of mitomycin analogs [102]. Treating hydroxylamine **127** with sodium hypochlorite effected the cycloaddition, but the wrong regioisomer **128** was obtained (Scheme 37). Steric repulsions between the methoxy ether and the olefin were probably responsible for this unexpected outcome. However, introduction of a carboethoxy group on the terminus of the olefin **129** restored the regioselectivity observed by Kozikowski to give oxazoline **130**.

#### 5.2. Reinhoudt. Rearrangement of 1-(1-pyrrolidinyl)-1,3-butadienes

**Scheme 38:**
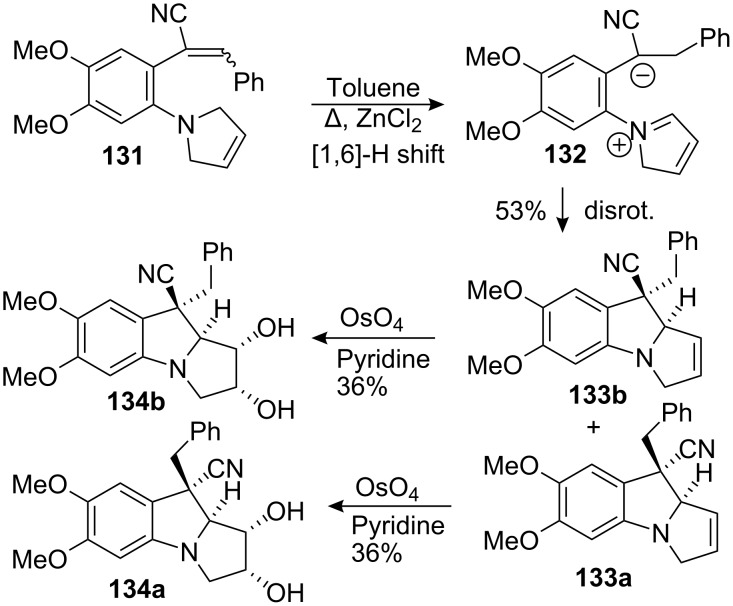
Synthesis of a mitosane core by rearrangement of a 1-(1-pyrrolidinyl)-1,3-butadiene.

Reinhoudt illustrated the stereoselective thermal rearrangement of 1-(1-pyrrolidinyl)-1,3-butadienes for the synthesis of mitosane analogs [103–105]. They proposed that the transformation proceeded through two consecutive pericyclic reactions (Scheme 38). Starting from compound **131**, a [1,6] hydrogen shift produced the conjugated 1,5-dipolar species **132** that subsequently underwent concerted disrotatory electrocyclisation of the 6π-electron system to give a mixture of the *cis* and *trans* isomers **133a** and **133b** (ratio 1.8:1). Compound **133b** was separated by chromatography and a final dihydroxylation using a stoichiometric quantity of osmium tetroxide gave mitosane **134b**, which resulted from attack on the convex face of the molecule. The same stereochemical outcome was observed using the other diastereomer **133a** showing that the substituents on C9 had only a minor influence on the stereoselectivity of the dihydroxylation.

Compounds **134a** and **134b** could be tailored as mitomycin A and mitomycin B derivatives respectively but issues pertaining to the introductions of the C9a oxygenated functionality and the carbamoyl moiety at C10 remain unsolved.

#### 5.3. Sulikowski. Buchwald coupling and carbene insertion

**Scheme 39:**
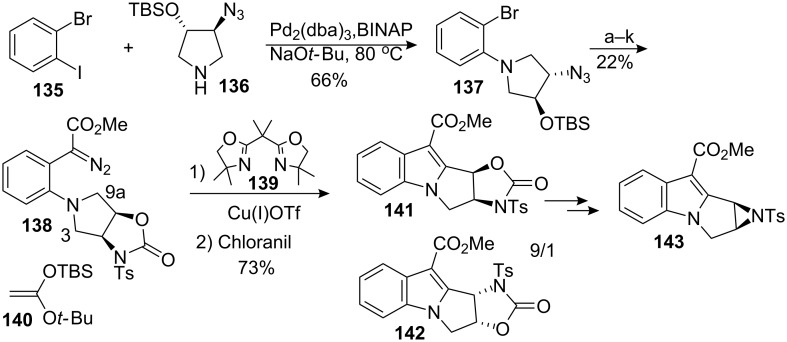
Sulikowski synthesis of an aziridinomitosene. ^a^Pd(Tol_3_P)_2_Cl_2_, Bu_3_SnF, **140**; ^b^H_2_, Pd/C; ^c^TFAA, Et_3_N; ^d^TBAF; ^e^MsCl, Et_3_N; ^f^DBU; ^g^K_2_CO_3_, MeOH; ^h^*p*-TsCl, Et_3_N; ^i^Cl_2_CO, Pyridine; ^j^*p*-TSA, MeOH; ^k^NaHMDS then PNBSA (para-nitrobenzenesulfonylazide).

The laboratory of G.A. Sulikowski proposed a synthesis of 1,2-aziridinomitosenes [106–107] using as key transformations a Buchwald–Hartwig cross-coupling [108–110] and a chemoselective intramolecular carbon-hydrogen metal-carbenoid insertion reaction (Scheme 39).

The chiral pyrolidine **136** was prepared in 94.7% *ee* by asymmetric ring opening of the corresponding N-Boc protected *meso* epoxide using the Jacobsen (salen)Cr(III) complex and TMSN_3_ [111]. The acetate group was installed on the aromatic using a palladium catalyzed reaction with the silylketene acetal **140** in the presence of tributyltin fluoride [112]. After protecting group manipulations and formation of the diazoester **138** with p-nitrobenzenesulfonylazide (PNBSA), the key carbene insertion was achieved using copper(I) in the presence of bis-oxazoline **139**. A 9:1 ratio of **141** and **142** was obtained after direct chloranil oxidation of the indoline intermediate to the indole. The regioselectivity is in accord with the results of Adams who showed that the C–H bond with the highest electron density was the most likely to migrate during rhodium(II) mediated C–H insertion [113].

**Scheme 40:**
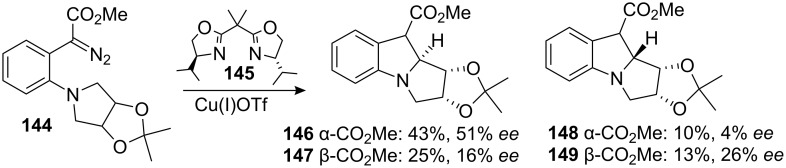
Enantioselective carbene insertion.

A related study assessed the possibility of circumventing this regioselectivity issue by exploiting the enantioselective intramolecular C–H insertion of diazoester **144** into a meso pyrrolidine using chiral catalyst **145**. Unfortunately the reaction displayed low enantio- and diastereoselectivity, with the major isomer **146** having an *ee* of only 51% (Scheme 40) [114].

#### 5.4. Parson. Radical cyclization

**Scheme 41:**
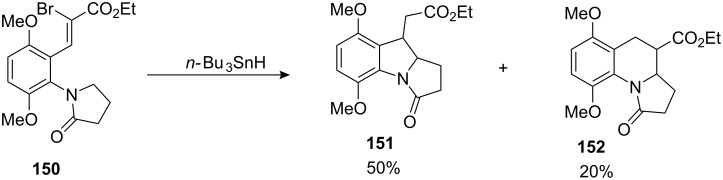
Parson’s radical cyclization.

The development of novel cascade (or domino) radical reactions is an active area of current research, and one approach to the mitomycin ring system focused on the application of 1-6-hydrogen atom transfer to create a pyrrolidinone radical, which could then undergo 5-*exo* cyclisation [115]. Following reaction of **150** with tributyltin hydride and AIBN, the desired 5-5-6-tricycle **151** was isolated in 50% yield as a 7.3:1 mixture of diastereoisomers (Scheme 41). Gratifyingly, the 6-*endo* product **152** was formed in only 20% yield and no simple reduced product was isolated.

This route allowed a convergent approach to the mitomycins via a tandem radical cyclisation process. It also provided an elegant approach to an intermediate pyrrolidinone radical, which proved impossible to access from a classical halogen-atom transfer route because of the difficulty in preparing the requisite 5-halopyrrolidinone precursor.

#### 5.5. Cha. Dialkoxytitanacyclopropane addition to imides

**Scheme 42:**
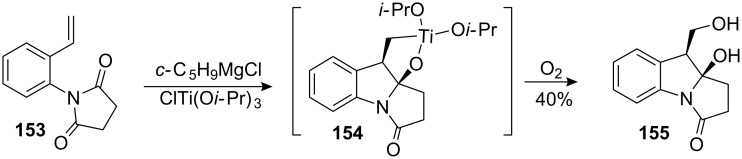
Cha’s mitomycin B core synthesis.

This methodology provides a very elegant way to install the C9a hydroxyl group, which remains the biggest challenge of mitomycin synthesis. Based on the precedent of ester cyclopropanation in presence of a titanocyclopropane developed by Kulinkovich [116], Cha’s approach to mitomycins involves the intramolecular addition of the same dialkoxytitanacyclopropane to an imide [117]. In contrast to the Kulinkovich’s cyclopropane synthesis, the imide proved to be resistant to cyclopropanation and the titanacycle intermediate **154** reacted further with electrophiles. Application of this methodology to imidostyrene **153** provided the mitomycin B core structure **155** in a reasonable 40% yield (Scheme 42).

Although this approach allowed the easy introduction of the C9a hydroxyl functionality, many problems remain unsolved: will the reaction remain efficient with a more functionalized substrate (*i.e.*, an electron rich aromatic, as necessary to introduce the quinone, and substituents at the C1 and C2 position, as needed for the later introduction of the aziridine) and, more importantly, will it be possible to conserve the extremely labile C9a hydroxyl group after removal of the stabilizing carbonyl at C3?

### 6. The N-aromatic disconnection

**Scheme 43:**
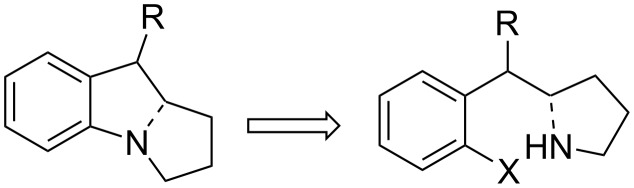
The N-aromatic disconnection.

#### 6.1. Kishi. Synthesis of mitomycin C

**Scheme 44:**
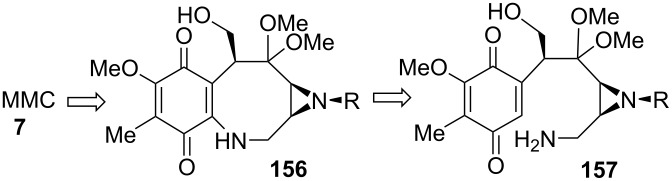
Kishi retrosynthesis.

This is the first total synthesis of a mitomycin and it surely constitutes a landmark in the field of organic chemistry. The retrosynthesis is extremely logical in the sense that Kishi noticed that the aminal moiety was the most sensitive part of the molecule [15–17]. Therefore, he introduced it at the very end of the synthesis. To do so, the transannular cyclisation of the methoxy-ketal derivative **156** was very appealing. The requisite eight membered ring was formed by the well-established chemistry of quinones using an intramolecular Michael addition by the primary amine **157** (Scheme 44) [118].

**Scheme 45:**
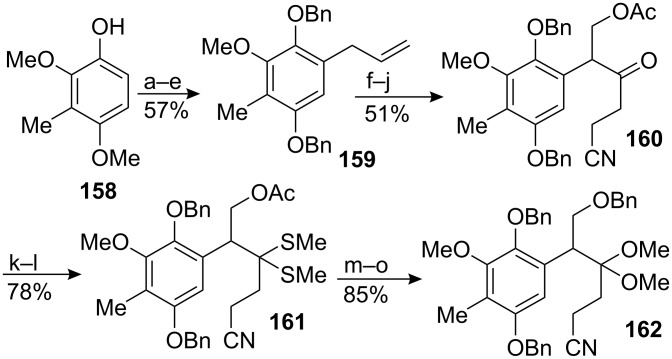
Kishi synthesis of a starting material. ^a^allyl bromide, K_2_CO_3_, acetone, reflux; ^b^N,N-Dimethylaniline, reflux; ^c^70% HNO_3_/AcOH; ^d^Zn, AcOH, 0 °C; ^e^BnBr, K_2_CO_3_, DME-DMF, reflux; ^f^H_2_O_2_, C_6_H_5_CN, K_2_CO_3_, MeOH-dioxane; ^g^LDA, MeCN, −30 °C; ^h^CrO_3_, H_2_SO_4_, water-acetone; ^i^MeONa, (CH_2_O)_3_, MeOH-THF, 0 °C; ^j^Ac_2_O, Py, 0 °C; ^k^MeSH, BF_3_ · 2AcOH, −30 °C; ^l^Et_3_N, MeOH, ^m^NaHCO_3_, MeOH-DCM; ^n^BnBr, KH, DMF; ^o^HgCl_2_, MeOH.

The synthesis began with the known phenol **158** which was reacted with allyl bromide to trigger a Claisen rearrangement (Scheme 45). This installed the three carbon chain on the newly formed para-methoxyphenol which was subsequently oxidized to the quinone then reduced to yield the corresponding *para*-catechol. This latter compound was protected with benzyl groups. Although this sequence of protecting group interconversion required many steps it was absolutely necessary for the success of the synthesis. Protection with benzyl groups has tremendous advantages in that they are both robust and easily removed under neutral conditions – necessary for removal without damage to the sensitive aziridine and ketal groups later in the synthesis. Moreover, the simultaneous deprotection of both benzyl groups leads to a hydroquinone which is readily oxidized into the quinone by simple exposure to oxygen. On the other hand, the oxidation of a para-methoxyphenol is more perilous since it would involve stronger oxidants that could damage other parts of the molecule. The synthesis followed Scheme 45 with a noteworthy methoxy-ketal formation going through the dithiane **161**.

**Scheme 46:**
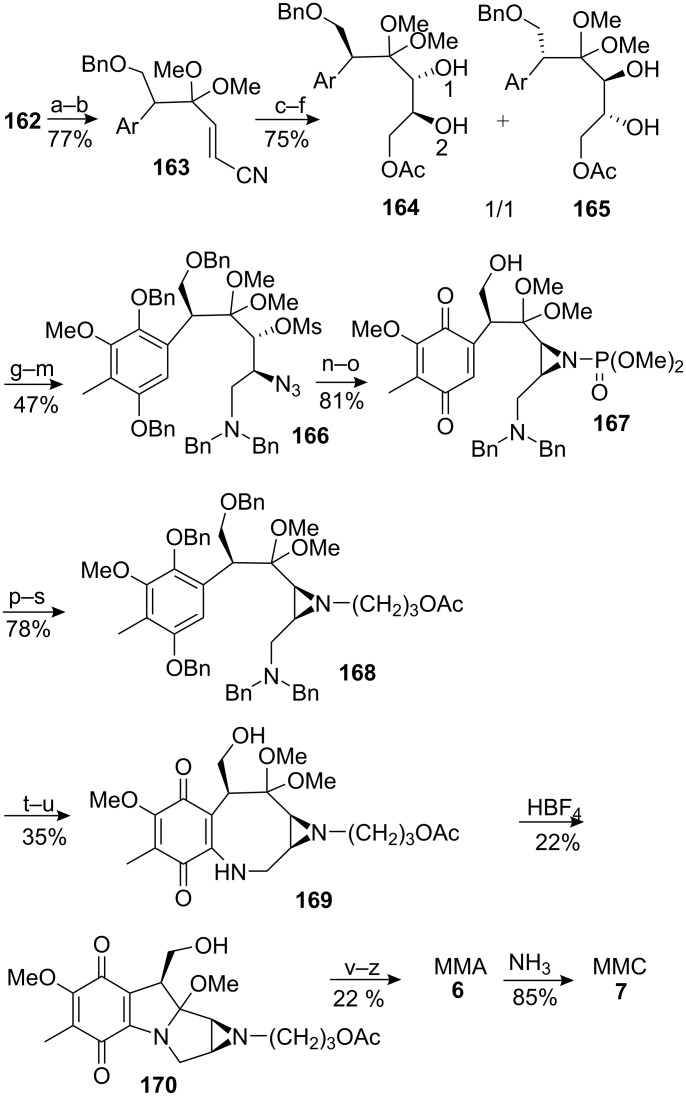
Kishi synthesis of MMC **7**. ^a^LDA, THF, −78 °C then PhSeBr, THF, −78 °C; ^b^H_2_O_2_, THF-EtOAc; ^c^DIBAL, DCM-Tol, 0 °C; ^d^NaBH_4_, MeOH-DCM, 0 °C; ^e^Ac_2_O, Py; ^f^OsO_4_, Py-THF. ^g^MsCl, Et_3_N, DCM, 0 °C; ^h^NaH, DMF; ^i^MeONa, MeOH-DCM; ^j^LiN_3_, DMF, 150 °C; ^k^Ms_2_O, Py; ^l^BnNH_2_, 150 °C; ^m^BnBr, K_2_CO_3_, acetone, reflux; ^n^(MeO)_3_P, reflux; ^o^NaH, THF; ^p^LAH, Et_2_O; ^q^CH_2_=CHCHO; ^r^B_2_H_6_, DCM; ^s^Ac_2_O, Pyr; ^t^H_2_, Pd-C, DCM; ^u^O_2_, MeOH; ^v^COCl_2_, N,N-dimethylaniline, DCM-Tol; ^w^NH_3_, DCM-Tol, 0 °C; ^x^NaOCH_3_, MeOH-DCM; ^y^DMSO-DCC, TFA-Py; ^z^HClO_4_, DCM.

The introduction of the aziridine was elaborated via a diol in the usual way [119]. However, three equivalents of osmium tetroxide and one week of reaction time were needed to get decent yield of the diasteromeric diols **164** and **165** from olefin **163** (Scheme 46). This is probably due to the poor reactivity of the olefin, which is considerably reduced by the inductive effects of the neighbouring ketal and acetate groups [120–121]. The diol **164** was isolated by chromatography and selectively mesylated on hydroxyl 2. Treatment with sodium hydride formed the epoxide. The acetate was removed and the epoxide was opened with lithium azide at 150°C. The two resulting alcohols were mesylated and the primary mesylate displaced with benzylamine. The resulting secondary amine was benzylated to give **166**. Reduction of the azide with trimethylphosphite followed by intramolecular S_N_2 displacement gave aziridine **167**. Due to the reactivity of the aziridine in mitomycin C, a special protecting group was used during the synthesis. The aziridine protecting phosphate group in **167** was cleaved using lithium aluminum hydride and replaced by a 3-acetyl propyl group to give **168**. This group was removed by hydrolysis, Pfitzner–Moffat oxidation [122] and retro-Michael (steps x, y, z). As discussed before, all the benzyl groups were removed at once to yield compound **169** after exposure to air.

**Scheme 47:**
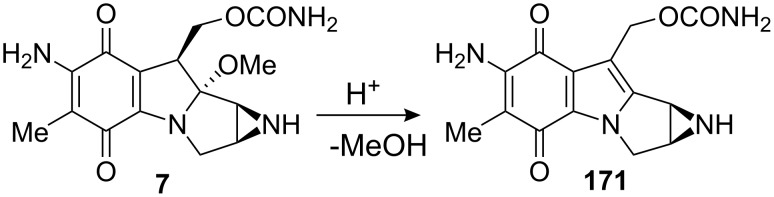
Acid catalyzed degradation of MMC **7**.

The key step was the trans-annulation using tetrafluoroboric acid as catalyst. It was expected that the product of this reaction would be fairly stable because of the decreased nucleophilicity of the pyrrole nitrogen in conjugation with the quinone ring. Mitomycin C itself is indeed only moderately reactive with acids before reduction of the quinone [35]. And the other hand, Boruah and Skibo have provided evidence that the loss of the C9a methoxy group to give aziridinomitosene **171** was specifically acid-catalyzed in mitomycin C (Scheme 47) [123], but thankfully the aminal functionality in compound **170** remained stable in the presence of tetrafluoroboric acid.

The end of the synthesis involved the introduction of the carbamoyl moiety by reaction of the primary alcohol with phosgene then ammonia. The protecting group of the aziridine was then removed to give mitomycin A, which was converted in mytomycin C by treatment with ammonia (Scheme 46). The Kishi synthesis was realized in 44 linear steps with a global yield of 0.16%.

#### 6.2. Kametani

**Scheme 48:**
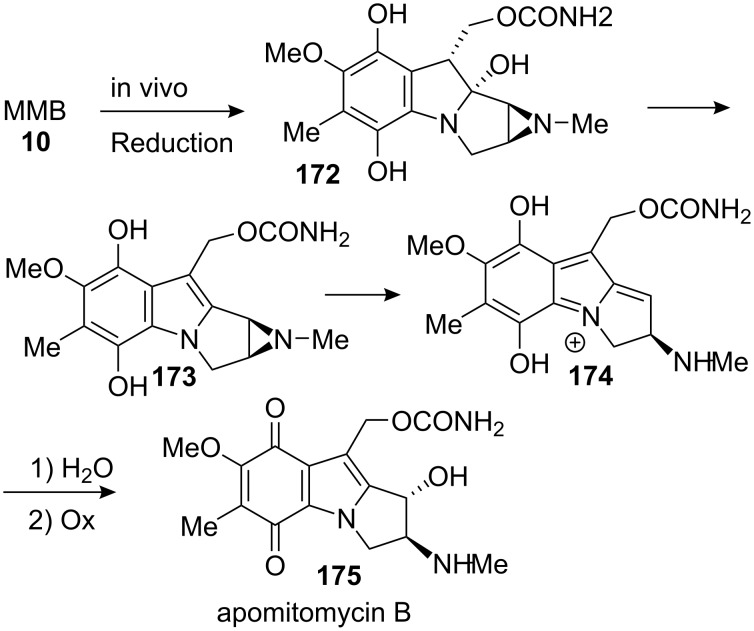
In vivo formation of apomitomycin B.

This methodology was investigated by T. Kametani in Japan in the late 1960’s and led to the formation of a derivative of apomitomycin B [124–128]. This compound comes from the metabolic hydrolysis of either mitomycin B, **10**, or mitomycin F, **8**, by the mechanism shown in Scheme 48 [129].

**Scheme 49:**
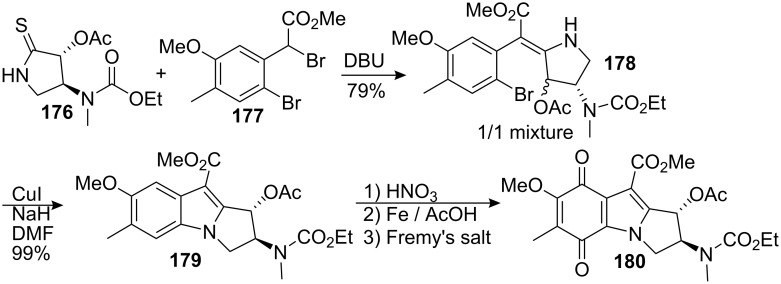
Advanced intermediate for apomitomycin B synthesis.

This compound has shown anti-tumour and anti-bacterial activities and thus is a valid target for a synthesis [85]. Being completed just after the period culminating in the synthesis of Vitamin B12 by the groups of Woodward and Eschenmoser [130], this synthesis utilized a powerful methodology developed during this period to make vinylogous amides [131–132]. Pyrrolidinethione **176** was reacted with the dibromoester **177** to give the *Z* isomer of the vinylogous carbamate **178** (Scheme 49). An erosion of the *trans* relationship between the two substituents of the pyrrolidine was observed giving a 1:1 mixture of *cis* and *trans* isomers. An intramolecular Buchwald coupling with copper iodide concluded the formation of the pyrrolo-indole tricycle. During this last step the *trans* relationship between the substituents of the pyrrolidine was restored to give the more stable isomer **179**.

The quinone ring was then elaborated by aromatic nitration of compound **179**, reduction to the aniline and subsequent treatment with Fremy’s salt to give compound **180**. No further developments were made on this substrate in part due to the difficulties encountered during reduction of the methyl ester [124]. It is known that the treatment of quinones with mild reducing agents gives hydroquinones while strong reductants modify the quinone in a less specific manner [133–134].

#### 6.3. Remers. Synthesis of a functionalized mitosene

**Scheme 50:**
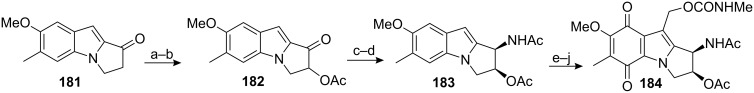
Remers synthesis of a functionalized mitosene. ^a^TMSCl, Et_3_N, ZnCl_2_ then NBS; ^b^AcOK; ^c^NH_2_OH; ^d^Pd/C, H_2_, Ac_2_O; ^e^POCl_3_, DMF; ^f^90% HNO_3_; ^g^Fe, AcOH; ^h^Fremy’s salt; ^i^NaBH_4_ then FeCl_3_; ^j^O=C=NMe, Et_3_N.

W.A. Remers used a similar approach to build the quinone ring of a 1,2-disubstituted mitosene [135]. The target compound had an arrangement of the C1 and C2 substituents opposite to that found in mitomycin solvolysis products. Unfortunately, these compounds did not show any anti-tumour activity [136]. Starting with the tricyclic ketone **181** [137–138] a low yielding bromination reaction was undertaken followed by an acetate displacement of the resulting α-bromoketone to give **182** (Scheme 50). The amine was introduced by formation of an oxime followed by catalytic hydrogenation in the presence of acetic anhydride to give the *cis* acetamido-acetate **183**.

The methylene side chain containing the methyl-carbamate was introduced via a Vilsmeier–Haack reaction and the aromatic ring was oxidized with the same method discussed in section 6.2 (vide supra). The synthesis was completed by selective sodium borohydride reduction of the remaining aldehyde, followed by alkylation of the resulting primary alcohol with methyl isocyanate to give **184**.

#### 6.4. Coleman. Allylation reaction and 1,4-quinone addition

**Scheme 51:**
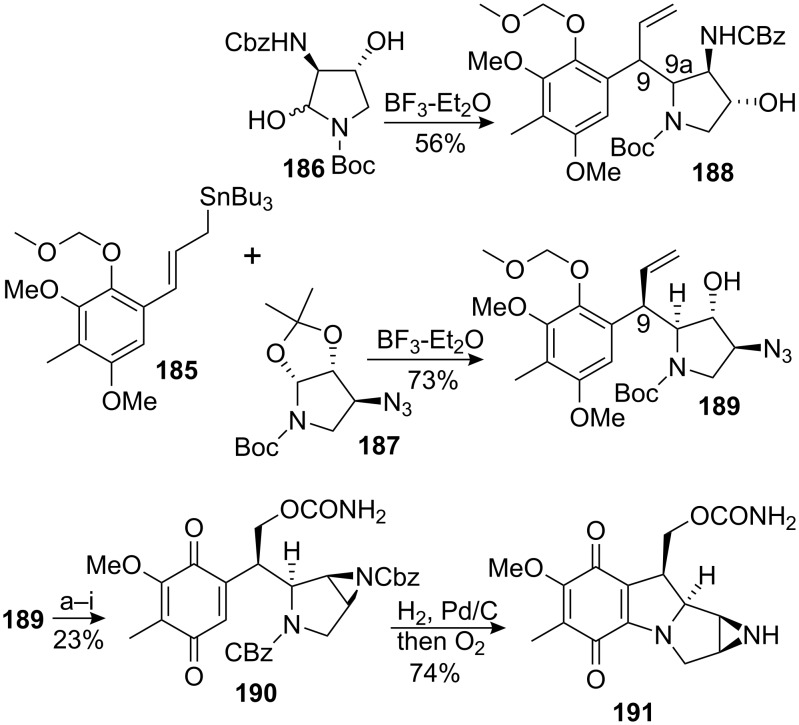
Coleman synthesis of desmethoxymitomycin A. ^a^SnCl_2_, PhSH, Et_3_N, CH_3_CN; ^b^ClCO_2_Bn, Et_3_N; ^c^PPh_3_, DIAD, THF; ^d^O_3_; ^e^NaBH_4_, MeOH; ^f^Me_3_SiOTf, 2,6-di-*tert*-butylpyridine, DCM; ^g^ClCO_2_Bn, Et_3_N; ^h^trichloroacetylisocyanate, DCM; ^i^CAN, CH_3_CN-H_2_O.

**Scheme 52:**
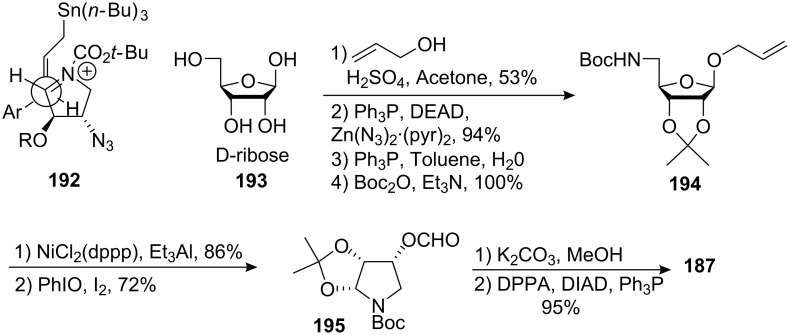
Transition state and pyrrolidine synthesis.

The Coleman group proposed an elegant synthesis of an enantiomerically pure mitosane. One of the key transformations involved an allylation reaction [139–143] between the allyl stannane **185** and the iminium formed in situ from the enantiomerically pure pyrrolidine **187** (Scheme 51) [144]. The reaction showed good diastereoselectivity, giving a 3:1 ratio of diastereomers favoring the desired isomer. Presumably, allylstannane **185** approached the iminium ion by a synclinal transition state **192** as shown in Scheme 52. The pro-*S* face of the iminium being less hindered and possessing a lower LUMO energy than the pro-*R* face, the approach occurred accordingly from the face opposite the alkoxy substituent. When the allylation reaction was done with the iminium derived from pyrrolidine **186**, no diastereoselectivity for the formation of **188** was observed. The bulkiness of the benzyl carbamate now proximal to the iminium ion was responsible for this poor result. The pyrrolidine **187** was synthesized from D-ribose **193**. Treatment of D-ribose in acetone with allylic alcohol in presence of catalytic amount of sulfuric acid provided the corresponding protected acetonide allyl glycoside. The primary alcohol was transformed into a N-Boc amine by successive Mitsunobu reaction, reduction and acylation to give compound **194**. The allyl group was cleaved using nickel chloride and triethylaluminium and fragmentation with iodosobenzene and iodine afforded compound **195**. The formyl group was hydrolyzed and the azide was introduced using diphenylphosphoryl azide (DPPA) to give **187**.

The azide **189** was then selectively reduced to the corresponding amine with stannous chloride and thiophenol and the aziridine was fashioned by protection of the primary amine followed by an intramolecular Mistunobu reaction. The tetracyclic framework was completed by an intramolecular Michael addition to give desmethoxymitomycin A **191**. Although the angular methoxy group at the C9a position is lacking in regards to natural mitomycin A, the authors claim that oxidation of the C9a position might still be possible, in accordance with a precedent in the literature [145].

**Scheme 53:**
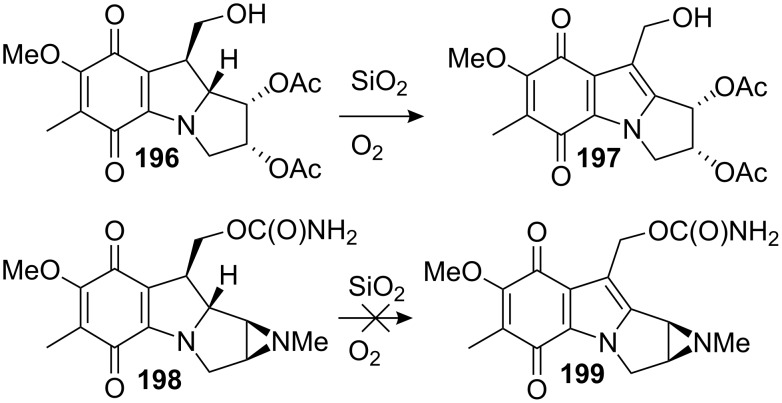
Air oxidation of mitosanes and aziridinomitosanes.

A direct C9a oxidative transformation from desmethoxymitomycin A is an interesting but difficult approach. Previous reports proved that the mitosanes are very sensitive molecules. In his studies directed towards the oxidation of an aziridinomitosane to an aziridinomitosene, Danishefsky found that the mitosane **196** underwent oxidative transformation to mitosene **197** upon simple exposure to silica gel in the presence of air (Scheme 53). On the other hand, attempting the same reaction with the aziridinomitosane **198** met with failure and only non-characterizable products were obtained. The presence of the aziridine was presumed to be responsible for this limitation [146]. The study showed that while mitomycins are stable to these conditions, their aziridinomitosane equivalents are not and proved that the C9a methoxy/hydroxyl group is important for the stability of mitomycins.

### 7. The C9-aromatic disconnection

**Scheme 54:**
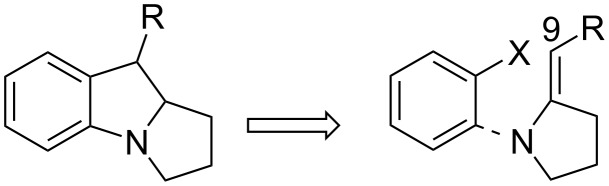
The C9-aromatic disconnection.

#### 7.1. Johnston. Enamine conjugate addition

**Scheme 55:**
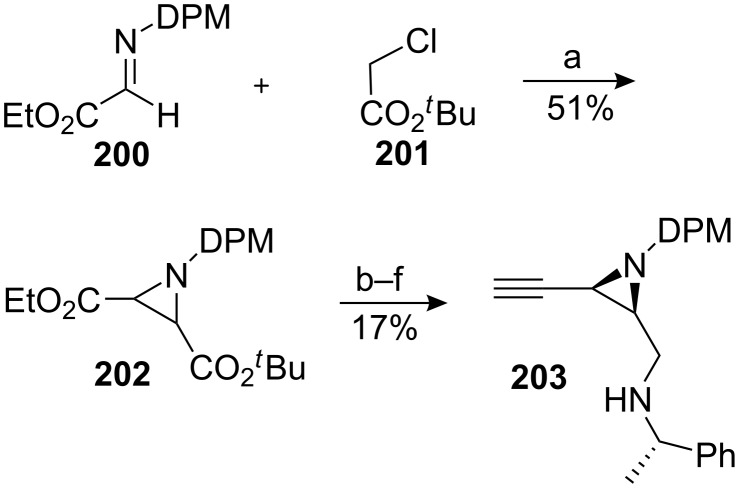
Synthesis of the aziridine precursor. ^a^LHMDS, THF; ^b^NaOH; ^c^(s)-α-Me-BnNH_2_, DCC, HOBT; ^d^DIBAL; ^e^K_2_CO_3_, Gilbert’s reagent [148]; ^f^Red-Al, 90 °C.

Johnston utilizes the natural negative polarity of the C9 carbon for the construction of an advanced intermediate en route to a mitomycin [147]. A Darzens reaction using the azomethine electrophile **200** provided an easy entry for the construction of the starting *cis* aziridine **202** which was converted in five steps to the alkynyl amine **203** (Scheme 55).

**Scheme 56:**
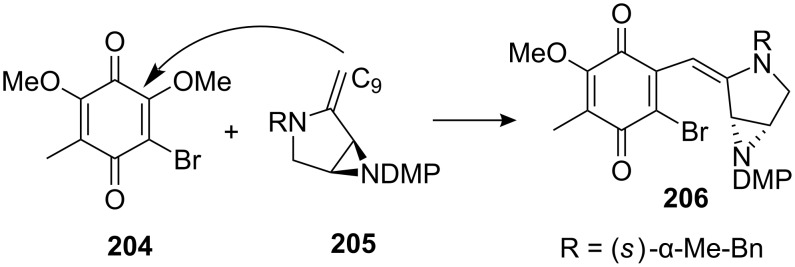
Synthesis of **206** via enamine conjugate addition.

An aminomercuration of compound **203** with an Hg(II) salt generated the enamine **205** which was reacted in situ with quinone **204**. The addition was regioselective for methoxy substitution at the bromomethoxy olefin and gave access to an advanced intermediate lacking only the C10 hydroxymethyl to complete the mitomycin carbon backbone (Scheme 56). However, compound **206** was very unstable with a half-life of 1.5 days at −15 °C.

#### 7.2. Rapoport. Intramolecular Heck coupling

**Scheme 57:**
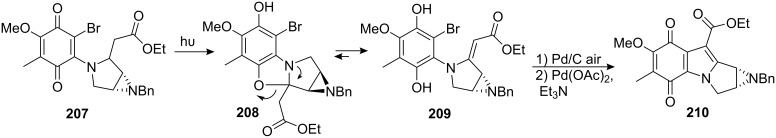
Rapoport synthesis of an aziridinomitosene.

In 1983 Rapoport released a fascinating synthesis of aziridinomitosenes based on a photochemical oxidation and an intramolecular Heck reaction [149]. Exposure of the quinone **207** to sunlight triggered the formation of benzoxazole **208**, which cleaved to form an intermediate iminium salt. Subsequent proton transfer gave the vinylogous carbamate **209** (Scheme 57). After oxidation of the hydroquinone to the quinone, a palladium catalyzed ring closure afforded the aziridinomitosene **210**.

**Scheme 58:**
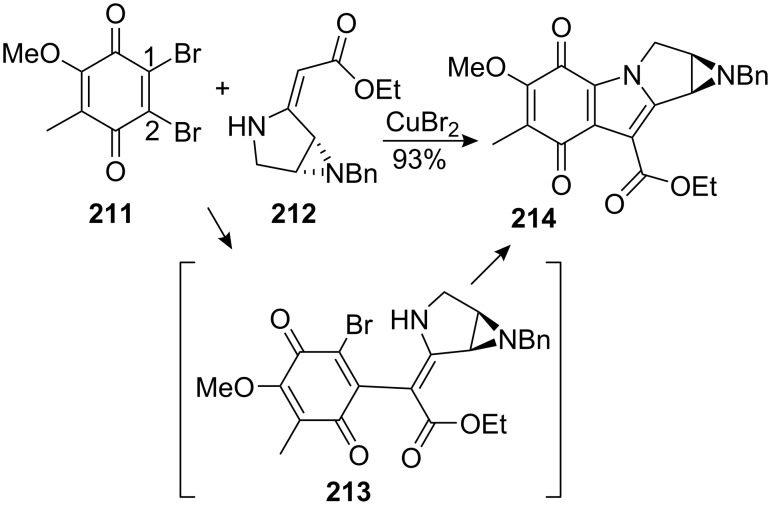
One pot synthesis of a mitomycin analog.

A more direct approach involved the addition of unsaturated aziridinopyrrolidine **212** to dibromoquinone **211** followed by cupric bromide catalyzed ring closure (Scheme 58). This one-step strategy unfortunately afforded the unnatural regioisomer **214** as the major product. Because carbon C2 in quinone **211** is more electrophilic than carbon C1, nucleophilic addition-elimination of vinylogous carbamate **212** at the carbon centre gave intermediate **213** which cyclized in situ to give compound **214**.

#### 7.3. Michael. Intramolecular Heck coupling

**Scheme 59:**
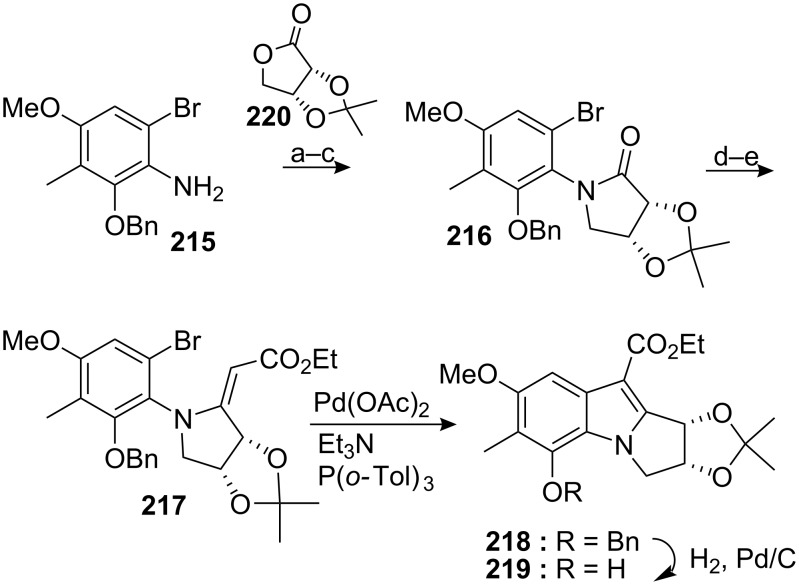
Synthesis of compound **218** via intramolecular Heck coupling. ^a^EtMgCl, THF, then **220**; ^b^MsCl, Et_3_N; ^c^NaH, THF-DMF; ^d^Lawesson’s reagent, toluene; ^e^BrCH_2_CO_2_Et, Zn, I_2_.

Recently the Michael group proposed a formal enantioselective asymmetric synthesis of an aziridinomitosene also based on an intramolecular Heck coupling [150]. They succeeded in incorporating all the reactive functionalities, namely the quinone, the carbamate and the aziridine and thus bypassed the challenging reduction of the robust C9 ester (found, for instance, in the Rapoport synthesis). Coupling of bromo aniline **215** with lactone **220** formed the lactam **216** in three steps with 90% yield (Scheme 59). This latter compound was transformed into the vinylogous carbamate **217** using a Reformatsky addition to the corresponding thiolactam. The crucial intramolecular Heck cyclisation was carried out with palladium acetate, tri-*o*-tolylphosphine and triethylamine to give **218** in 82% yield.

**Scheme 60:**
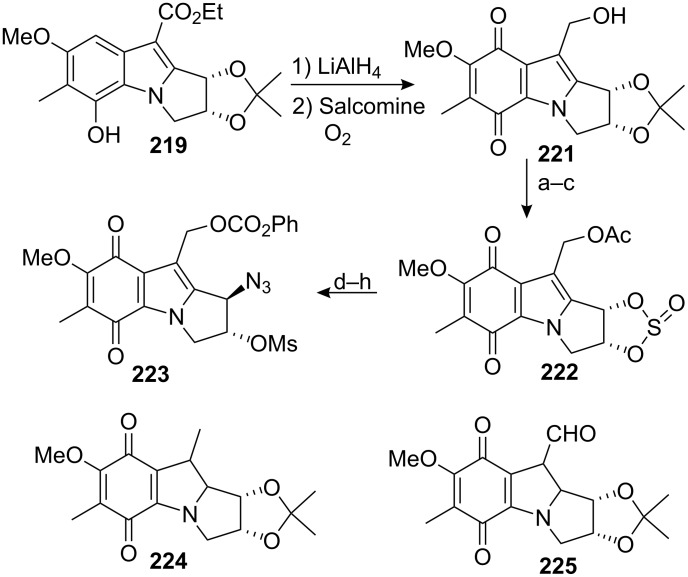
Elaboration of indole **223**. ^a^Et_3_N, Ac_2_O; ^b^AcOH; ^c^SOCl_2_, Et_3_N; ^d^NaN_3_, DMF; ^e^H_2_SO_4_, THF; ^f^K_2_CO_3_, MeOH; ^g^PhOCOCl, pyridine; ^h^MsCl, Et_3_N.

It was discovered that transformation of compound **218** into an aziridinomitosane was unexpectedly difficult. The most successful approach involved deprotection of the benzyl ether to generate the corresponding phenol **219** followed by reduction of the ester with lithium aluminium hydride and direct oxidation of the phenol to the quinone **221** with molecular oxygen and a catalytic amount of salcomine in an overall 30% yield over the three steps (Scheme 60). However many problems arose during this process, including over-reduction of the ester to the alkane **224** and the oxidation of the alcohol to the aldehyde **225** during the quinone oxidation step.

The synthesis was completed by protection of the primary alcohol with an acetyl group and by deprotection of the ketal with acetic acid. The resultant diol was reacted with thionyl chloride and the corresponding sulfite **222** was opened with sodium azide; oxidation to the more reactive sulfate was not necessary. The final sequence involved deprotection of the acetate followed by acylation of the more reactive primary alcohol with phenyl chloroformate and subsequent mesylation of the secondary alcohol to give **223**, whose spectroscopic data agreed with those reported by Jimenez [151].

### 8. From indoles

**Scheme 61:**
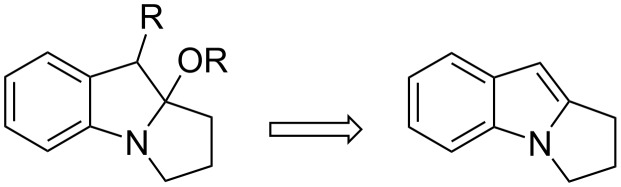
C9-C9a functionalization from indole.

#### 8.1. Jimenez. Synthesis of mitomycin K

**Scheme 62:**
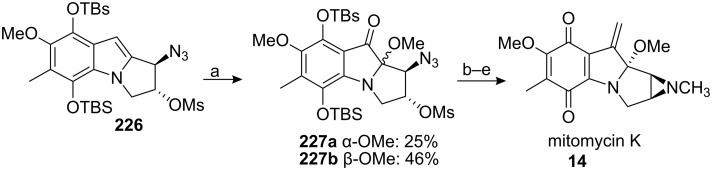
Synthesis of mitomycin K. ^a^2 equiv. MoO_5_.HMPA, MeOH; ^b^PPh_3_, Et_3_N, THF-H_2_O; ^c^MeOTf, Py, DCM; ^d^Me_3_SiCH_2_Li, THF; ^e^PCC.

The idea of converting an indole to a mitomycin is appealing since it simplifies considerably the retrosynthetic pathway. Using this idea, Jimenez was able to affect the direct oxidation of indole **226** with (hexamethylphosphoramido)oxodiperoxomolybdenum (VI) (MoO_5_ • HMPA) to give diastereomers **227a** and **227b**, which were readily elaborated into mitomycin K (Scheme 62) [20,152].

**Scheme 63:**
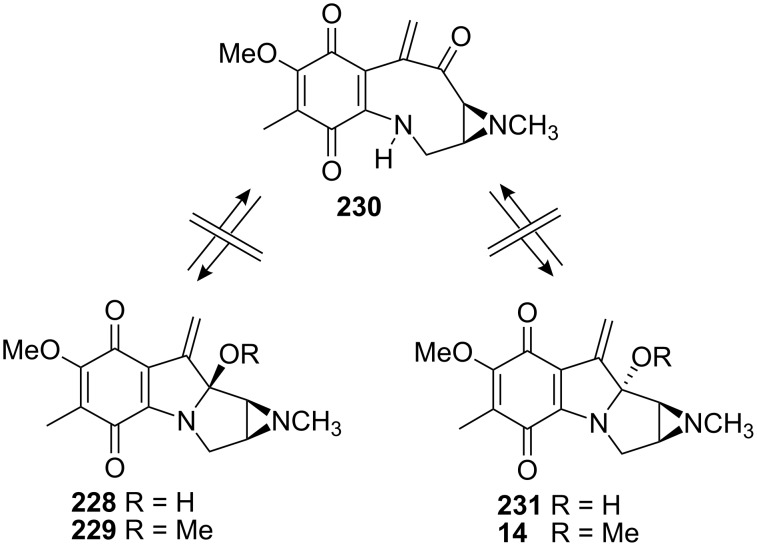
Configurational stability of mitomycin K derivatives.

The critical indole oxidation step gave a selectivity of about 2:1 in favor of the undesired isomer **227b** (β−OMe) but fortunately this compound could be epimerized at the C9a carbon using 0.2M HCl in MeOH to give a 1:1 ratio of the 2 diastereoisomers **227a** and **227b**. The Danishefsky group had observed an extraordinary configurational stability of the C9a carbon in *epi*-mitomycin K [146]. *Epi*-mitomycin K **229** or its demethylated derivative **228** treated under various acidic or basic conditions were constantly recovered intact (Scheme 63). They rationalized this phenomenon by the incapacity of those compounds to open to the carbinolamine intermediate **230** in contrary to what was observed for the mitomycin B series (Scheme 3) [27].

**Scheme 64:**
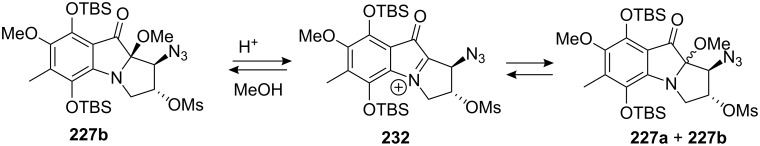
Epimerization of carbon C9a in compound **227b**.

For compound **227b**, we can infer that the aromatic nitrogen receives significant enhancement of its nucleophilic character compared to compound **229** because of the presence of the TBS protected hydroquinone. Consequently, the formation of the reactive iminium species **232** under acid catalyzed conditions can lead to epimerization of the C9a centre (Scheme 64).

**Scheme 65:**
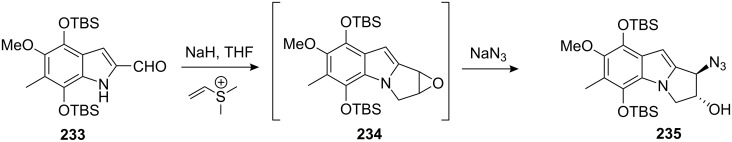
Corey–Chaykovsky synthesis of indol **235**.

The overall yield for the 13 step synthesis was 1.4%. The elegant synthesis of the starting material **235** used an intramolecular Corey–Chaykovsky reaction. The anion of indole **233** was reacted with dimethylvinylsulfonium iodide to give the transient epoxide **234** which was opened in situ by the addition of sodium azide to give azido-alcohol **235** (Scheme 65).

**Scheme 66:**
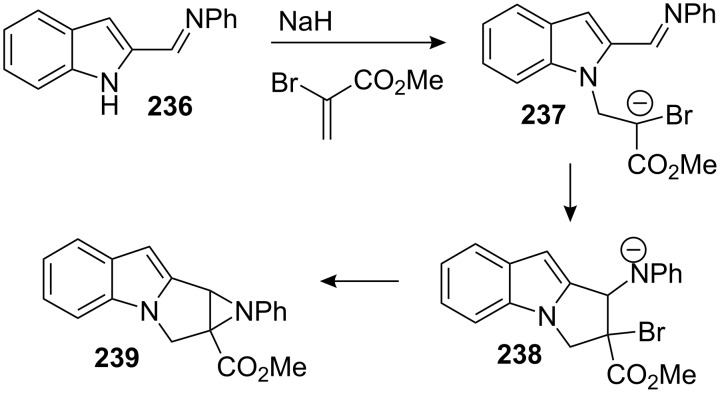
Cory intramolecular aza-Darzens reaction for the formation of aziridinomitosene **239**.

Before this work, Cory used a similar strategy involving an intramolecular aza-Darzens reaction to give directly the aziridinomitosene analogue **239** (Scheme 66) [153]. Treatment of 2-(*N*-phenylformimidoyl)indole **236** with sodium hydride followed by addition of methyl 2-bromopropenoate gave the aziridinomitosene **239**. The reaction presumably proceeded through intermediates **237** and **238**.

#### 8.2. Jimenez. Synthesis of an aziridinomitosene

**Scheme 67:**
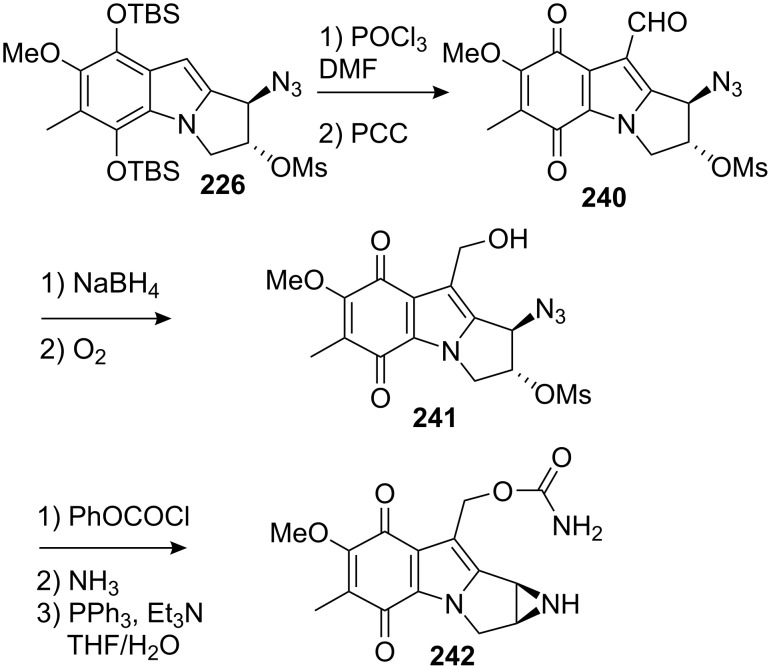
Jimenez synthesis of aziridinomitosene **242**.

A few years later, Jimenez and co-workers were able to transform the azido mesylate indole **226** into a fully functionalized mitosene [151]. Formylation of the mitosene **226** using a Vilsmeyer–Haack reaction followed by oxidative cleavage of the TBS groups with PCC gave the quinone **240** in 80% yield (Scheme 67). Reduction of the aldehyde with sodium borohydride followed by reoxidation of the hydroquinone by bubbling oxygen into the reaction mixture provided the alcohol **241** in 74% yield. This latter material was transformed to the aziridinomitosene **242** in a three step sequence in 53% yield.

The synthesis of the fully functionalized aziridinomitosene **242** was accomplished in 16 steps from 2,5-dimethylanisole in 3.4% overall yield.

#### 8.3. Kametani. Von Braun opening of indoline

**Scheme 68:**
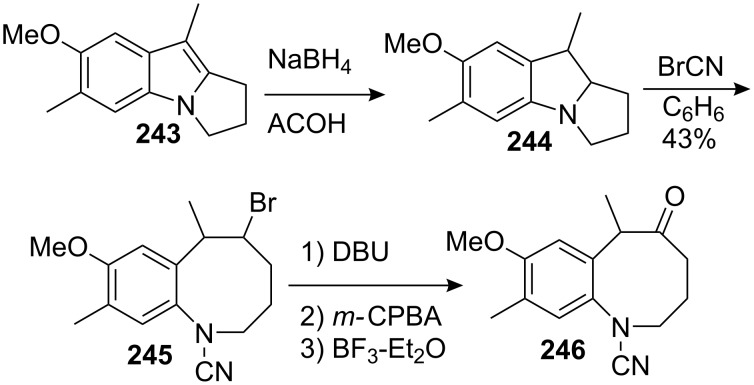
Von Braun opening of indoline **244**.

Kametani found another alternative for the elaboration of an advanced intermediate for the synthesis of mitomycins using indoles as starting materials [154–155]. Pyrrolo[1,2-a]indole **243** was reduced with sodium borohydride in acetic acid [156]. The resulting indoline **244** was opened using a Von Braun reaction with cyanogen bromide to give **245**. The direct Kornblum oxidation [157] of this compound with dimethylsulfoxide and sodium bicarbonate at 150 °C gave only a few percent yield of the desired ketone. Therefore, the authors turned to a three step synthesis involving elimination, epoxidation and epoxide rearrangement mediated by boron trifluoride to give the desired benzazocin-5-one **246** [158–159] (Scheme 68). The transannular cyclisation of an eight membered ring ketone such as **246** seems a promising approach considering the Kishi’s successful synthesis (see above). The access of such structures is usually difficult, often requiring a long and poor yielding synthesis. The benefit of this method is that the pyrrolo[1,2-a]indoles **243** are easily available [160–162] and thus can be regarded as good precursors.

Although the overall yield of this process is not very high, it offers the possibility of accessing the benzazocinone substructures very quickly.

#### 8.4. Danishefsky. Oxidation of a leucoaziridinomitosane

**Scheme 69:**
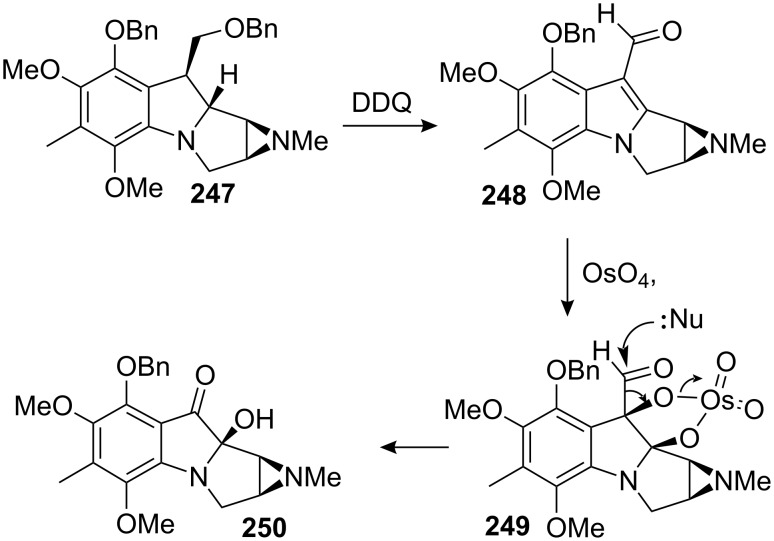
C9a oxidation of an aziridinomitosane with DDQ/OsO_4_.

Danishefsky and co-workers were very active in the field of the mitomycins and discovered an effective way to oxidize leucoaziridinomitosane **247** to leucoaziridinomitosene **248** using DDQ (Scheme 69) [146]. The unusual stability of this leucoaziridinomitosene certainly accrues from the C10 aldehyde which attenuates the nucleophilicity of the indolic nitrogen. Based on precedents [163–164], osmylation of the indole with a large excess of osmium tetroxide in pyridine over a few days gave the deformylated product **250** where the hydroxyl group arose *cis* to the aziridine. This result was surprising since the osmylation was expected to occur on the convex face of the molecule (i.e., *anti* to the aziridine). The authors claimed that the stereochemical outcome of the reaction was directed by the nitrogen of the aziridine, which, they reasoned, should be a better ligand for the osmium reagent than the vinylogous amide nitrogen of the indole.

**Scheme 70:**
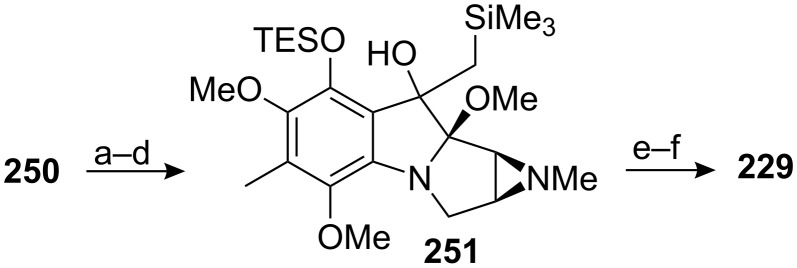
Synthesis of *epi*-mitomycin K. ^a^NaH, Me_2_SO_4_; ^b^H_2_, Pd/C; ^c^Mitscher reagent [165]; ^d^[(trimethylsilyl)methyl]lithium; ^e^TBAF; ^f^DDQ.

The synthesis of *epi*-mitomycin K was completed by methylation of the tertiary alcohol **250**, conversion of the benzyl protecting group on the phenol to a triethylsilyl group, Peterson olefination, deprotection of the triethylsilyl group and oxidation to quinone **229** (Scheme 70). This unnatural mitomycin possessed the opposite C9a configuration compared to mitomycin K and isomerisation by basic or acidic treatment was ineffective (Scheme 63).

### 9. Miscellaneous

#### 9.1. Fukuyama. Synthesis of mitomycin C via the mitomycin rearrangement

**Scheme 71:**
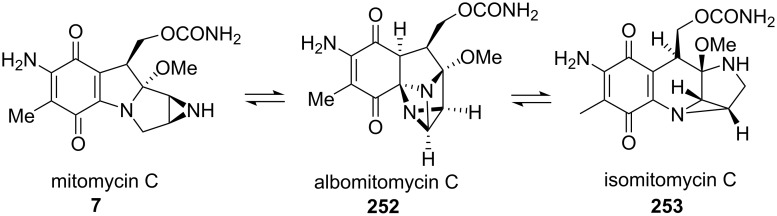
Mitomycins rearrangement.

Fukuyama’s approach to the mitomycins took into account the fact that these molecules can rearrange to give isomeric compounds called “isomitomycins” [26]. Mitomycin C can therefore equilibrate with “isomitomycin C” **253**, going through an isolable species **252** called “albomitomycin C” (Scheme 71). This transformation, called the “mitomycins rearrangement,” seemingly occurrs via a Michael, retro-Michael mechanism. The equilibrium favors the mitomycin form.

**Scheme 72:**
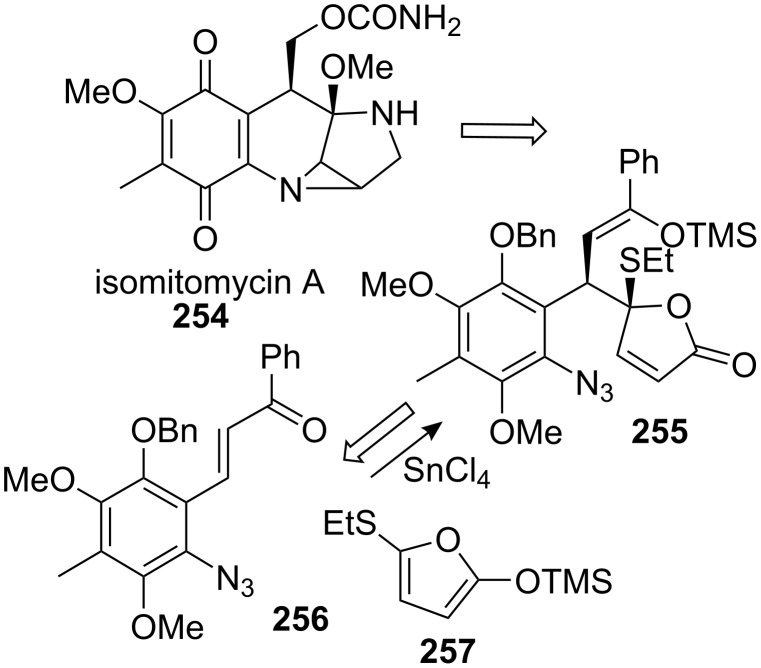
Fukuyama’s retrosynthesis.

This discovery led to an innovative strategy for the synthesis of mitomycin C [18,166]. There are tremendous advantages to using isomitomycin as a target since the elimination of methanol at C9a, the most problematic issue in mitomycin synthesis, is no longer a threat in isomitomycin because of its bridgehead position. Having isomitomycin A as a target, the corresponding tetracyclic structure was constructed by an intramolecular cycloaddition of the azide of compound **255** with the olefin of the five-membered lactone. Compound **255** was easily prepared via a Mukaiyama reaction of the silylenol ether **257** with the readily available chalcone **256** in 95% yield (Scheme 72). Although this reaction was racemic, other asymmetric versions of this reaction were recently studied, [167–171] but further attempts to achieve asymmetric synthesis proved unsuccessful using this route. Also, questions arose as to whether compound **255** was formed through a Mukaiyama reaction or through a Lewis acid (stannyl chloride) promoted Diels–Alder reaction favoring the endo addition. No direct evidence was produced to distinguish between the two reactions but the unusually high stereoselectivity observed might support the Diels–Alder reaction mode.

**Scheme 73:**
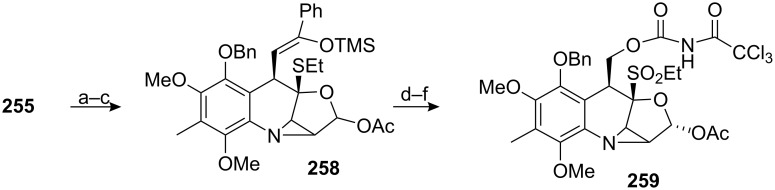
[2+3] Cycloaddition en route to isomitomycin A. ^a^Toluene, 110 °C; ^b^DIBAL, THF, −78 °C; ^c^Ac_2_O, Py.; ^d^RuO_2_, NaIO_4_, EtOAc/H_2_O (1/1); ^e^NaBH_4_, MeOH; ^f^CCl_3_CONCO, DCM.

Heating compound **255** in toluene triggered a [2+3] cycloaddition between the azide and the lactone olefin. The intermediate triazoline was not observed and the aziridine was directly obtained in 86% yield (Scheme 73). A subsequent reduction of the lactone with DIBAL gave the corresponding lactol, which was protected with an acetyl group to give **258**.

The silyl enol ether of **258** was then cleaved using ruthenium tetroxide generated in situ by the Sharpless methodology [172]. Although the aldehyde formed during the course of this reaction is usually over-oxidized into a carboxylic acid, in this specific case it seemed that the steric hindrance surrounding the aldehyde prevented the formation of the gem-diol mandatory for further oxidation. Nonetheless, the thiol was oxidized into the corresponding sulfone.

**Scheme 74:**
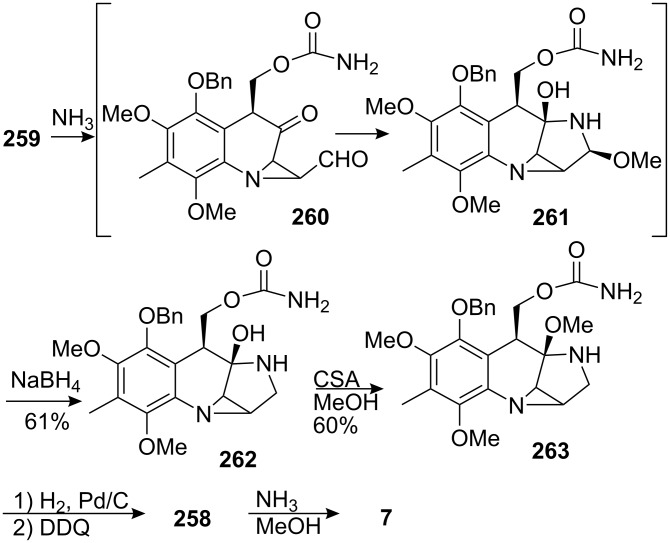
Final steps of Fukuyama’s synthesis.

The aldehyde was then reduced and transformed into the trichloroacetyl carbamate **259** by treatment with trichloroacetyl isocyanate. Subsequent treatment with ammonia triggered a series of transformations that led to compound **261** (Scheme 74). Under these conditions, the trichloroacetyl carbamate was cleaved as well as the acetate, which furnished **260** after loss of a molecule of ethane sulfinic acid. The formation of **261** occured most likely by addition of a molecule of methanol to the corresponding imine. Compound **261** was not isolated because of stability issues but was directly reduced with sodium borohydride to give **262**. The bridged hemiaminal of **262** was not reduced because it would involve the formation of an extremely strained bridgehead double bond. Accordingly, the authors used a strong acid (camphorsulfonic acid, CSA) in methanol to enable iminium formation and subsequent transformation to the methoxy aminal **263**. The aromatic ring was then oxidized into the corresponding quinone by hydrogenolysis of the benzyl group followed by DDQ oxidation to give isomitomycin A, **254**. A final treatment with ammonia gave mitomycin C **7**.

Ten years after the Kishi synthesis, Fukuyama realized the second and last racemic total synthesis of mitomycin C with an overall yield of 10%.

#### 9.2. Ban–Shibasaki. “Crisscross” annulation

**Scheme 75:**
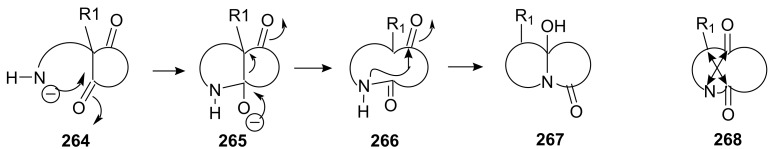
“Crisscross annulation”.

The rapid synthesis of medium-sized heterocycles has been the main research focus of Y. Ban since the beginning of the 1980’s. The “crisscross annulation” involves intramolecular nucleophilic addition of the nitrogen in β-diketone **264** followed by a retro-aldol ring opening to give keto-amide **266** before a final transannular cyclisation to give the “crisscrossed” product **267** in a one pot process (Scheme 75) [173–174].

**Scheme 76:**
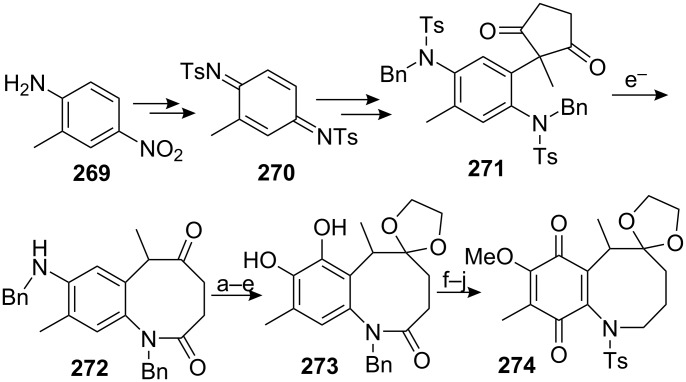
Synthesis of **274**; the 8-membered ring **274** was made using a crisscross annulation. ^a^20% Pd(OH)_2_/C, H_2_, AcOEt; ^b^Pb(OAc)_4_, DCM; ^c^HClO_4_, THF, DCM; ^d^10% Pd/C, H_2_, AcOEt; ^e^TMSCl, HOCH_2_CH_2_OH, MeOH, DCM; ^f^Me_2_SO_4_, K_2_CO_3_, CHCl_3_; ^g^LAH, THF; ^h^20% Pd(OH)_2_/C, H_2_, MeOH; ^I^TsCl, Py, DCM; ^j^Salcomine/O_2_, DMF.

**Scheme 77:**
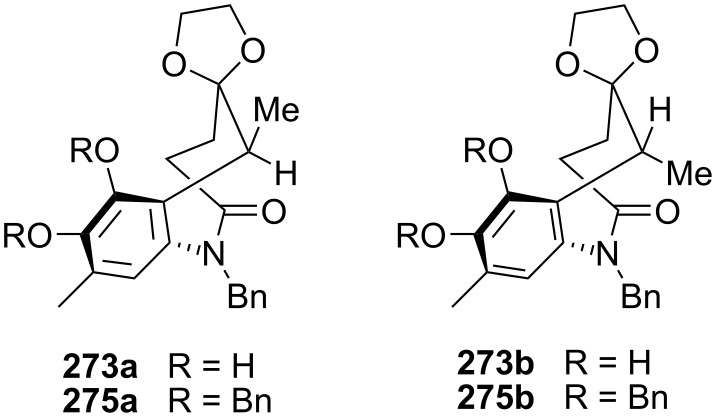
Conformational analysis of compound **273** and **275**.

This scheme was successfully employed by the same group for the synthesis of a decarbamoyloxymitomycin derivative [175–176]. Compound **271** was obtained readily from commercially available 2-amino-5-nitrotoluene **269** in 94% overall yield. An electrochemical removal of the tosyl group triggered the crisscross annulation cascade reaction to afford **272** (Scheme 76). A long sequence of reactions then allowed the oxidation of the aromatic ring into the desired quinone: first, a selective deprotection of the benzyl aniline with the Pearlman catalyst left the less reactive benzylamide untouched [177]. The resultant primary aniline was then oxidized using Pb(OAc)_4_ to give the corresponding *o*-quinone imide. Hydrolysis of this crude material with perchloric acid followed by hydrogenation provided the corresponding *ortho*-hydroquinone before 1,3-dioxolane protection of the ketone gave compound **273** in 48% overall yield. Interestingly, this compound appeared as a pair of conformational diastereoisomers, **273a** and **273b**, that could be separated by chromatography (Scheme 77). The free energy of activation for interconversion was estimated to be as high as 25 kcal · mol^−1^ at 25 °C.

Detailed study by NMR and X-ray crystallography showed that both of the two compounds **273a** and **273b** adopt a twist-boat-chair conformation (usually more energetic than the boat-boat conformation) in which the C6 methyl group is either in a pseudoequatorial (**273a**) or pseudoaxial position (**273b**) [178]. Further studies aimed at converting one isomer into the other showed that **273a** was, as expected, the thermodynamically favoured compound (**273b** converts to **273a** after refluxing in benzene for 2 days). More interestingly, when a mixture of di-benzylated derivatives **275a** and **275b** were left at room temperature, isomer **275a** slowly isomerizes to **275b**. This special feature was attributed to the severe steric interactions between the C8 benzyloxy and C9 methyl groups in **275a**.

The catechol **273** was then turned into the desired quinone **274** by selective methylation of the phenol at C7 (the phenol at C8 being hindered by the C9 methyl group), reduction of the lactam with lithium aluminium hydride, and hydrogenolysis to give an unstable aminophenol which was directly tosylated. Finally, oxidation with bis(salicylidene)ethylenediiminocobalt(II) (salcomine) [179–180] provided the *p-*quinone **274** in 94% yield. The key step towards the elaboration of a mitomycin was the introduction of the aziridine at the benzazocenol stage, namely on the allylic alcohol **276**.

**Scheme 78:**
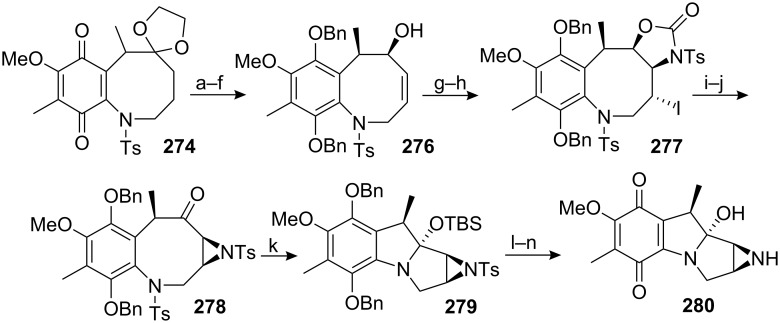
Synthesis of a mitomycin analog. ^a^Na_2_S_2_O_4_, H_2_O, DCM; ^b^BnBr (10 equiv), K_2_CO_3_, 18-crown-6 (cat.), THF, reflux; ^c^conc. HCI, THF, 0 °C; ^d^PhSeCl, 10% HCl (cat.), AcOEt; ^e^NaIO_4_, H_2_O, THF; ^f^DIBAL, THF, −70 °C; ^g^Ts-N=C=O, THF; ^h^I_2_, K_2_CO_3_, THF; ^i^K_2_CO_3_, MeOH-DCM (1/2); ^j^PCC (33 equiv.), DCM; ^k^TBSOTf (2 equiv), NEt_3_, DCM, −78 °C; ^l^10% Pd/C, H_2_, NEt_3_, AcOEt; then O_2_; ^m^Na-naphthalene, THF, −98 °C; then O_2_; ^n^TBAF, AcOH, THF.

To this end, quinone **274** was reduced with sodium thiosulfate to the corresponding hydroquinone and bis-protected with benzyl bromide (Scheme 78). The ketal group was cleaved with concentrated hydrochloric acid before a selenium oxidation gave the corresponding benzazocenone. This sequence proceeded in a good overall yield of 65% and, surprisingly, the addition of PhSeCl in acidic medium occurred selectively on the less hindered alpha carbon of the benzazocin ketone. The authors did not comment on this unusual selectivity but one can imagine that the formation of an enol on the C9 side of the molecule would greatly enhance the steric effects between the C9 methyl group and the C8 benzyloxy group since the carbons C8, C8a, C9 and the methyl would become coplanar. A stereoselective 1,2 reduction of the enone with DIBAL gave the allylic alcohol **276**. All attempts to introduce the aziridine via the typical epoxidation or dihydroxylation and displacement with sodium azide failed. Therefore an intramolecular sequence was pursued. The allylic alcohol **276** was converted to the corresponding allylic carbamate by treatment with tosyl isocyanate. Activation of the olefin with iodine provided the cyclic carbamate **277** [181]. Hydrolysis of the carbamate using potassium carbonate in methanol was readily achieved due to the presence of an electron withdrawing tosyl group on the nitrogen. This process released a free sulfonamide that cyclized in situ by S_N_2 displacement to give the aziridine with complete stereoselectivity. The homobenzylic alcohol was then oxidized using PCC to give compound **278**.

Having the ketone **278** in hand, the authors’ next step was to affect the trans-annular cyclisation to construct the mitomycin skeleton. Whether or not this transformation should occur at the stage of the protected hydroquinone or at the quinone stage frequently arises in the debate about the stability of leucoaziridinomitosenes. Although the more reasonable pathway would be the transformation at the quinone stage following the Kishi procedure, the design of the synthesis and especially the presence of a tosyl group on the aniline prevented such a scheme. The removal of the tosyl group at the quinone stage would indeed most likely interfere with the other functionalities present in the molecule. Therefore, the trans-annular cyclisation was attempted at the stage of the protected hydroquinone.

Ketone **278** displayed a rather low IR absorption at 1700 cm^−1^, indicating the presence of a transannular effect with the N4 nitrogen. Therefore, an increase of the electrophilic character of the ketone would increase this transannular interaction until it reached a point where a new bond could be formed, causing loss of the tosyl group. The use of TBSOTf effectively activated the ketone towards this cyclisation to give **279** in a highly stereoselective manner. It is noteworthy that the conditions used in this reaction were mild enough for the leucoaziridinomitosane structure to be unaffected. The same reaction using TMSOTf also gave the cyclised product, but led to decomposition during the isolation process. Hydrogenolysis of **279**, followed by treatment with oxygen afforded the corresponding quinone, which was subsequently treated with sodium-naphthalene to remove the tosyl group on the aziridine in low yield (16%). The removal of the tosyl group at the quinone stage was probably the biggest limitation of the synthesis since one-electron reducing agents were shown to generate a reactive species that suffered elimination of the C9a heteroatom functionality [43], which could explain the low yield of this reaction. The final compound **280** was obtained by deprotection of the tertiary alcohol by treatment with TBAF and acetic acid.

#### 9.3. Vedejs. Intramolecular [3+2] cycloaddition

**Scheme 79:**
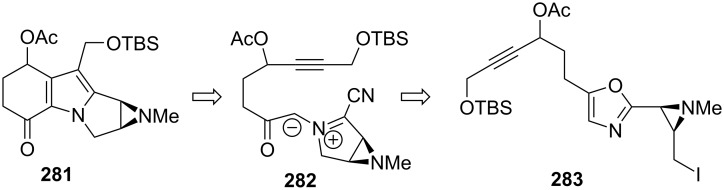
Vedejs retrosynthesis.

Vedejs developed a very innovative way to construct mitosenes, as he departed from the usual practice of forming the quinone by oxidation of an aromatic, as had been done in all previous syntheses [182–183]. This is surely an advantage since it allows manipulation of the labile leucoaziridinomitosenes at advanced synthetic stages to be avoided. His approach uses an intramolecular [3+2] cycloaddition between an azomethine ylide and an alkyne to assemble the tetracyclic aziridinomitosene core. The sequence began with conversion of oxazole **283** into the azomethine ylide **282** (Scheme 79).

**Scheme 80:**
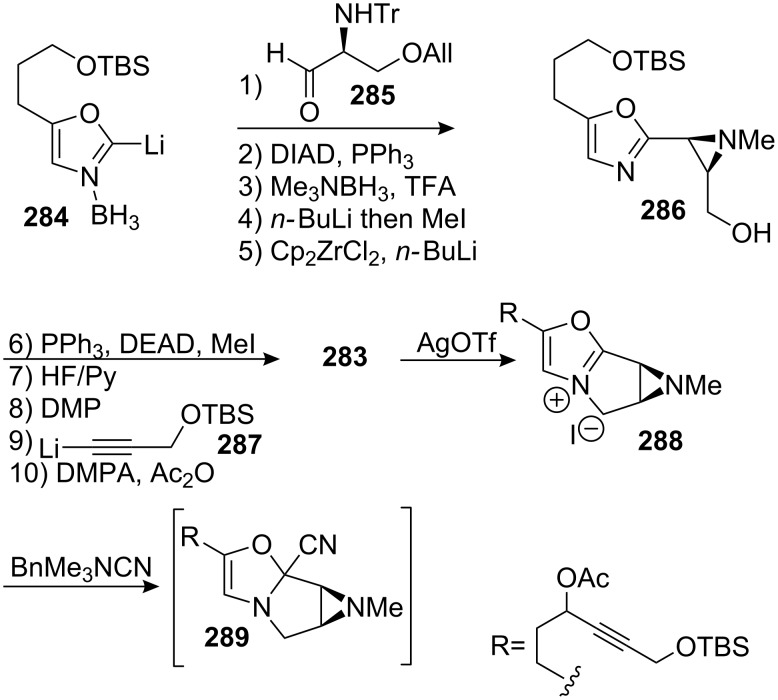
Formation of the azomethine ylide.

The azomethine ylide was prepared in 12 steps starting from the lithiated oxazole **284**. After reaction with the protected serinal **285**, a Mitsunobu reaction was used to install the aziridine (Scheme 80). The trityl protecting group was replaced with a methyl, and the O-allyl was removed using a low valent zirconium species generated in situ [184]. A few manipulations led to oxazole **283** which cyclized to **288** in the presence of silver triflate. The resulting salt was treated with a soluble source of cyanide (benzyltrimethylammonium cyanide, BnMe_3_NCN) which triggered the opening of the oxazole and the formation of the azomethine ylide **282**, probably through intermediate **289**. This compound reacted with the internal alkyne to give **281**, the tetracyclic core of the mitomycins.

**Scheme 81:**
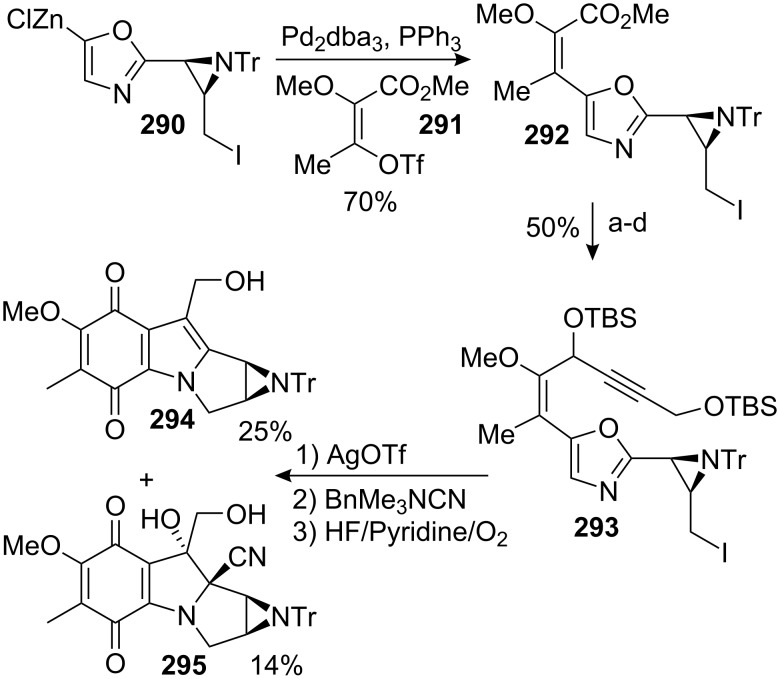
Vedejs second synthesis of an aziridinomitosene. ^a^DIBAL; ^b^TPAP, NMO; ^c^**287**; ^d^TBSCl, imidazole.

This route was suitable for the synthesis of aziridinomitosenes lacking the C7 methoxy group. Continuing efforts by the Vedejs group made possible the extension of this methodology to allow access to the fully functionalized quinone core found in mitomycin A [185]. A Negishi coupling of compound **290** with vinyl triflate **291** generated the oxazole **292** which was transformed in four steps into the alkyne **293** (Scheme 81). A similar activation cascade leading to the dipolar cycloaddition produced compound **294** after oxidation of the intermediate with a mixture of HF/pyridine/O_2_. A fair three-step yield of 25% was obtained, considering the dramatic changes in structure. The main isolated impurity of this process was compound **295**, arising from oxidation of the intermediate without elimination of hydrogen cyanide.

**Scheme 82:**
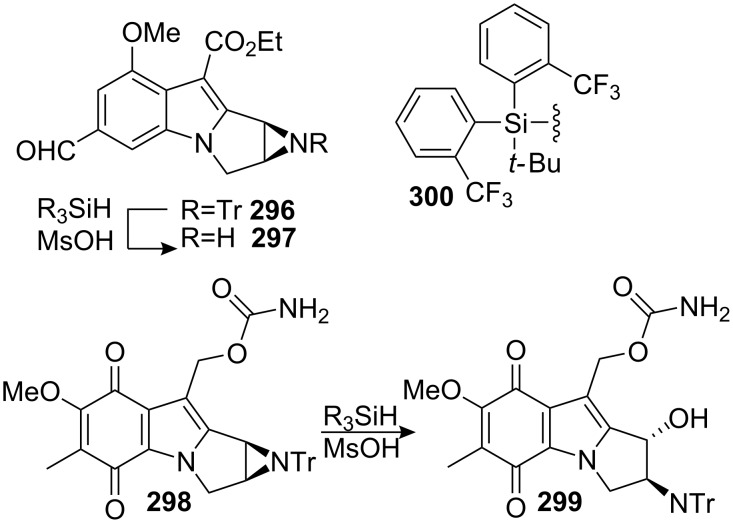
Trityl deprotection and new aziridine protecting group **300**.

Having compound **294** in hand, the target aziridinomitosene A could potentially be obtained by installation of the carbamate on the primary alcohol and removal of the trityl protecting group. In the hope that the vinylogous amide in **298** would behave similarly to the vinylogous carbamate in compound **296** that was successfully detritylated using methanesulfonic acid and triethylsilane, the same conditions were employed on compound **298**; unfortunately, only solvolysis product **299** was isolated (Scheme 82). Manifestly, the carbonyl of the quinone in **298** does not provide the same deactivating effect on the indole nitrogen as the carbonyl of the ester in **296**, making the aziridine of compound **298** more labile. Efforts to find a suitable protecting group for this reactive aziridine that could survive all the previous steps in the synthesis but, more importantly, could easily be removed at a late stage using non-acidic conditions led to the identification of the bulky silyl group **300** [186].

#### 9.4. Ciufolini. Homo-Brook rearrangement

**Scheme 83:**
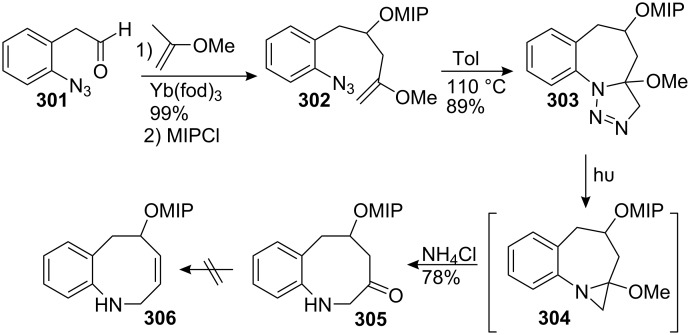
Ene reaction towards benzazocinones.

Recently Ciufolini reported a highly innovative and sophisticated retrosynthetic analysis for the construction of benzazocenols **306** which made possible the synthesis of FR-66979 [187]. Early studies aimed at the rapid synthesis of benzazacinones of type **305** identified the sequence depicted in Scheme 83 as a very efficient strategy. However, benzazocinone **305** could not be converted into the desired benzazocenol **306** which meant a dead end for the synthesis [188–189].

**Scheme 84:**
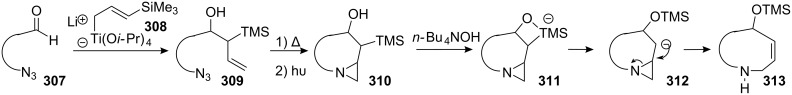
Benzazocenols via homo-Brook rearrangement.

A thorough analysis of the molecule suggested that an even more concise avenue to benzazocenols **306** might be possible by fragmentation of a silylated aziridine, followed by homo-Brook rearrangement [190]. The synthesis started by the addition of organometallic species **308**, developed by Reetz [191], to the azidoaldehyde **307**, which was followed by thermal azide cycloaddition and photochemical nitrogen extrusion to form the aziridine **310** (Scheme 84). Treatment of this compound with tetrabutylammonium hydroxide triggered a homo-Brook rearrangement, giving benzazocenol **313** in only four steps from aldehyde **307**.

This represents one of the most efficient sequences for the assembly of benzazocenols, even compared with those involving olefin metathesis. The synthesis of mitomycins using this methodology is currently being investigated.

#### 9.5. Iwasawa. Pt(II) or Au(II) catalyzed [3+2] cycloaddition of azomethine ylides

**Scheme 85:**
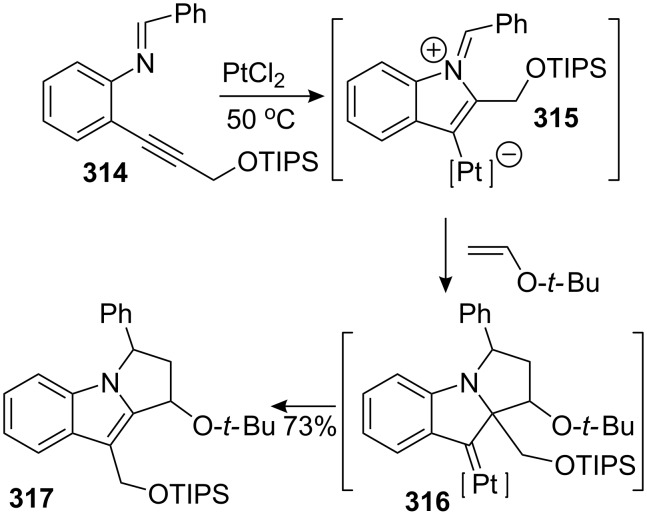
Pt-catalyzed [3+2] cycloaddition.

In 2006, this group disclosed the use of third row transition metals to catalyze efficiently the one-pot formation of pyrroloindoles starting from monocyclic materials. A number of transition metals have emerged as efficient catalysts for the electrophilic activation of alkynes in the past decades [192], and this group exploited such methodology by activation of the internal alkyne moiety in **314** to induce nucleophilic attack of the imino nitrogen, generating the corresponding metal-containing azomethine ylide **315** via 5-*endo* cyclisation (Scheme 85). Successive [3+2] cycloaddition and 1,2-alkyl migration gave the mitosene **317**, bearing a silyloxymethyl group in the C9 position, in 73% yield.

This sequence appears to be a very efficient way to generate mitosenes but many questions remain unsolved for the possible synthesis of mitomycins: is it possible to find an alkyl group at the imine of the starting material that can be easily removed at the end of the reaction? How to affect oxidation at the C2 position for the later introduction of the aziridine? Will the reaction work with the electron rich aromatic needed for conversion to the quinone?

#### 9.6. Trost. Pd-catalyzed carbonylative lactamization

**Scheme 86:**
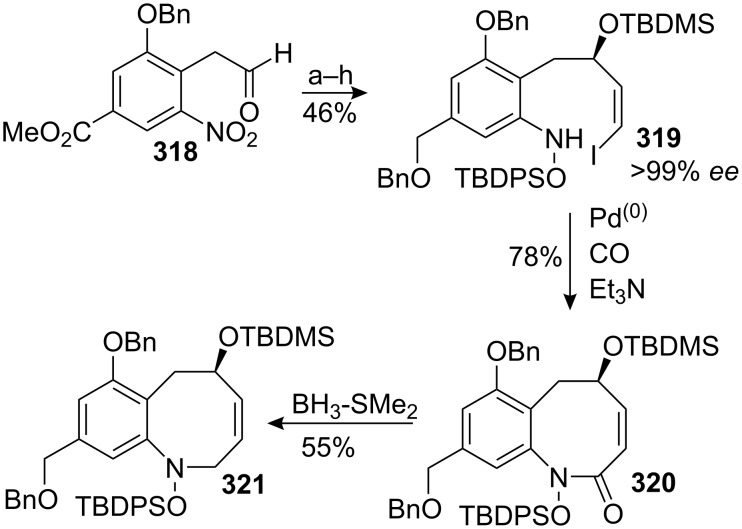
Carbonylative lactamization entry to benzazocenols. ^a^Zn(OTf)_2_, (+)-*N*-methylephedrine, Et_3_N, TMS-acetylene; ^b^DIBAL; ^c^K_2_CO_3_, MeOH; ^d^NaH, BnBr; ^e^NIS, 10% AgNO_3_; ^f^dipotassium azodicarboxylate, pyridine, AcOH, MeOH; ^g^SmI_2_; ^h^TBDPSCl, imidazole.

Although this study was mainly directed towards the synthesis of FR-900482, it provides an easy entry to enantiopure benzazocenol structures. Based on Carreira’s asymmetric acetylide addition to aldehydes in the presence of zinc (II) triflate and (+)-*N*-methylephedrine [193], the addition of trimethylsilyl acetylene into aldehyde **318** gave the corresponding propargylic alcohol with very high ee. Subsequent transformations led to the (*Z*) olefin **319** which cyclised in presence of palladium and carbon monoxide to give the eight membered lactam **320** (Scheme 86). The lactam was reduced with borane-methyl sulfide complex without competing with 1,4-reduction or cleavage of the N–O bond to provide the benzazocenol core found in FR-900482.

This synthetic sequence seems suitable for the synthesis of both FR-900482 and the mitomycins, although difficulties may arise from the introduction of the hydroxymethyl side-chain on C9 in the mitomycin series as previously experienced by Williams (see paragraph 3.3).

#### 9.7. Metathesis

Olefin metathesis has been known since the 1960’s, but it was not until the early 1990’s that this transformation became an important tool in synthetic organic chemistry. Since then a number of elegant applications of RCM (ring closure metathesis) in the total synthesis of heterocycles have been recorded [194].

#### Miller’s approach

**Scheme 87:**
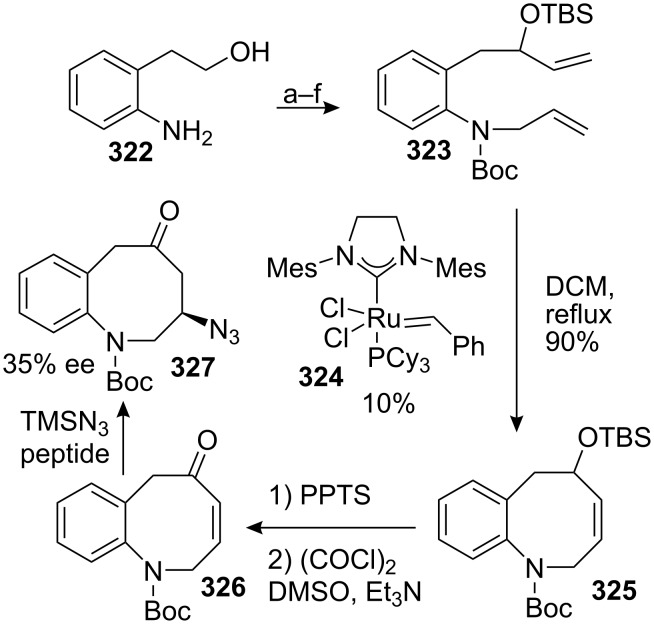
8 membered ring formation by RCM. ^a^BOC_2_O, NaHCO_3_; ^b^TBSCl, Imidazole, DMF; ^c^allyl bromide, NaH, DMF; ^d^PPTS, MeOH; ^e^(COCl)_2_, DMSO, Et_3_N, then vinyl magnesium bromide, −78 °C; ^f^TBSCl, imidazole, DMF.

The construction of a benzazocenone of type **326** could provide access to mitomycin derivatives. Miller’s group was interested in this approach for the synthesis of mitosene analogs [195]. The alcohol **322** was easily elaborated into benzazocenone **326** through a metathesis reaction using Grubbs II catalyst **324** (Scheme 87). Oxidation to the enone allowed introduction of the aziridine by enantioselective peptide-mediated conjugate addition of azide [196]. However this reaction proved to be inefficient, as enantiomeric excesses of no more than 35% were recorded for compound **327**. In general, enone **326** proved to be sluggish towards conjugate additions. This comes from the fact that the carbonyl group and the olefin are twisted 100.4° out of conjugation.

**Scheme 88:**
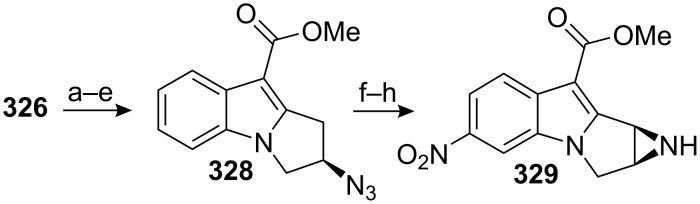
Aziridinomitosene synthesis. ^a^TMSN_3_; ^b^TFA; ^c^POCl_3_, DMF; ^d^NaClO_2_, NaH_2_PO_4_, 2-methyl-2-butene; ^e^MeI, Hünig’s base; ^f^Cu(NO_3_)_2_, Ac_2_O; ^g^NBS; ^h^PPh_3_, H_2_O-THF.

Regardless, synthesis of the mitosene core could be achieved by transannular cyclisation, formylation/oxidation, nitration, bromination and aziridine formation to give **329** (Scheme 88).

#### Pérez–Castells’ approach

**Scheme 89:**
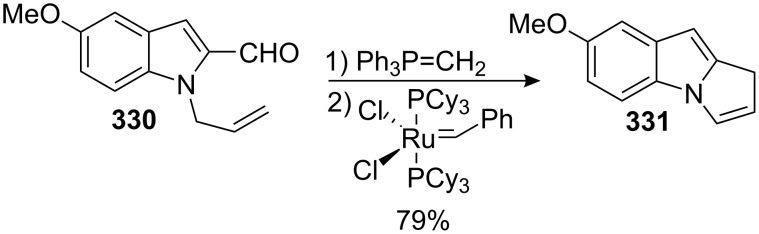
Metathesis from an indole.

The syntheses of pyrrolo-indoles has been attempted using many different methodologies, but Castells was the first to report the use of RCM starting from an indole [197]. Readily available aldehyde **330** was subjected to an efficient cascade Wittig-metathesis reaction to give compound **331**, where the double bond emerging from the metathesis reaction had shifted towards the indole nitrogen (Scheme 89).

Unfortunately, this migration moderated the utility of this methodology since it compromised efficient introduction of the aziridine at the C1–C2 position.

#### 9.8. Williams biosynthetic approach

**Scheme 90:**
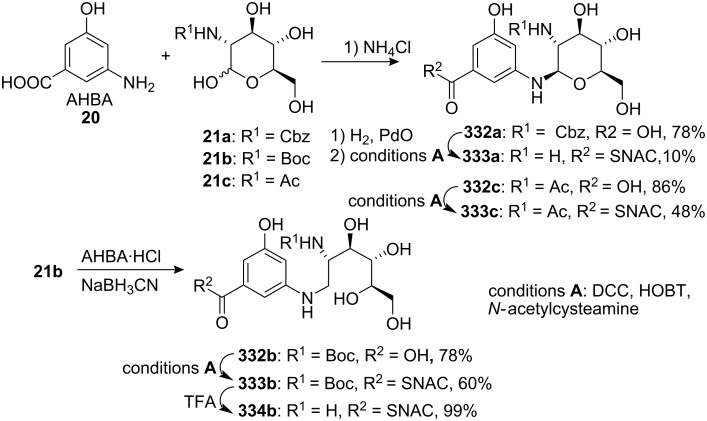
Synthesis of early biosynthetic intermediates of mitomycins.

In section 2.1 of this review, we described the two building blocks AHBA and D-glucosamine involved in the biosyntheses of mitomycins. Although a large amount of information was gathered on the genes and enzymes involved in the biosynthetic process, speculative assertions have arisen on how those two simple building blocks were assembled into the complex architectures of mitomycins [28]. In a recent paper, the Williams group described the synthesis of potential early biosynthetic intermediates to the mitomycins [198]. The compounds **332a** and **332c** were obtained as the β-anomer by condensation of AHBA with protected D-glucosamine **21a** and **21c** respectively (Scheme 90). The N-Cbz protected amine **332a** was unmasked using catalytic hydrogenolysis and the carboxylic acid was transformed to the *N*-acetylcysteamine (SNAC) thioester analog **333a**. The SNAC derivative of compound **332c** was obtained in a synthetically useful yield using the same procedure. The other putative biosynthetic intermediate equivalent **334b** was synthesized by reductive amination of AHBA hydrochloride with **21b**, followed by SNAC derivatization and unmasking the *N*-Boc protected amine with trifluoroacetic acid. Intermediates **332a**, **332c**, **333c**, **334b** could give valuable information about the biosynthesis pathway of mitomycins and provide fundamental understanding of biosynthetic enzymes implicated in the pathway. The success of combinatorial biosynthesis often depends on this later factor and the availability of the genetic information [199]. Many of the genes responsible for the biosynthesis of mitomycins being already identified, better drugs could be synthesized in the future through genetic engineering [200].

## Conclusion

Even 50 years after their discovery and 30 years after first submitting to total synthesis, the mitomycins are still regarded as challenging synthetic targets, and a practical synthetic route has yet to be discovered. Because mitomycin C is a valuable anti-cancer drug, thorough medicinal chemistry investigation, which would be abetted by the knowledge and material generated during a total synthesis, is still required. Such knowledge may well lead to new anti-cancer agents with a broad spectrum and potent activity.

## References

[1] Kudo S, Marumo T, Tomioka T, Kato H, Fujimoto Y (1958). Antibiot Chemother.

[2] Webb J S, Cosalich D B, Mowat J H, Patrick J B, Broschard R W, Meyor W E, Williams R P, Wolf C F, Fulmor W, Pidacks C (1962). J Am Chem Soc.

[3] Yudin A K (2006). Aziridines and Epoxides in Organic Synthesis.

[4] Hata T, Koga F, Sano Y, Kanamori K, Matsumae A, Sugawara R, Hoshi T, Shimi T, Ito S, Tomizawa S (1954). J Antibiot.

[5] Nagaoka K, Matsumoto M, Oono J, Yokoi K, Ishizeki S, Nakashima T (1986). J Antibiot.

[6] Kiyoto S, Shibata T, Yamashita M, Komori T, Okuhara M, Terano H, Kohsaka M, Aoki H, Imanaka H J (1987). J Antibiot.

[7] Hanada M, Ohkuma H, Yonemoto T, Tomita K, Ohbayashi M, Kamei H, Miyaki T, Konishi M, Kawaguchi H, Forenza S (1991). J Antibiot.

[8] Tsuchida T, Inuma H, Kinoshita N, Ikeda T, Sawa T, Hamada M, Takeuchi T (1995). J Antibiot.

[9] Wolkenberg S E, Boger D L (2002). Chem Rev.

[10] Coleman R S, Burk C H, Navarro A, Brueggemeier R W, Diaz-Cruz E S (2002). Org Lett.

[11] Alcaro S, Ortuso F, Coleman R S (2002). J Med Chem.

[12] Zein N, Solomon W, Colson K L, Schroeder D R (1995). Biochemistry.

[13] Casely-Hayford M A, Pors K, James C H, Patterson L H, Hartley J A, Searcey M (2005). Org Biomol Chem.

[14] Naoe Y, Inami M, Matsumoto S, Nishigaki F, Tsujimoto S, Kawamura I, Miyayasu K, Manda T, Shimomura K (1998). Cancer Chemother Pharmacol.

[15] Kishi Y (1979). J Nat Prod.

[16] Nakatsubo F, Fukuyama T, Cocuzza A J, Kishi Y (1977). J Am Chem Soc.

[17] Fukuyama T, Nakatsubo F, Cocuzza A J, Kishi Y (1977). Tetrahedron Lett.

[18] Fukuyama T, Yang L (1987). J Am Chem Soc.

[19] Benbow J W, McClure K F, Danishesky S J (1993). J Am Chem Soc.

[20] Wang Z, Jimenez L S (1996). Tetrahedron Lett.

[21] Mao Y, Varoglu M, Sherman D H (1999). J Bacteriol.

[22] Bradner W T (2001). Cancer Treat Rev.

[23] Lefemine D V, Dann M, Barbatschi F, Hausmann W K, Zbinovsky V, Monnikendam P, Adam J, Bohonos N (1962). J Am Chem Soc.

[24] Uzu K, Harada Y, Wakiki S (1964). Agric Biol Chem.

[25] Urakawa C, Nakano K (1981). J Antibiot.

[26] Kono M, Saitoh Y, Shirahata K, Arai Y, Ishii S (1987). J Am Chem Soc.

[27] Hornemann U, Heinz M J (1985). J Org Chem.

[28] Grüschow S, Chang L C, Mao Y, Sherman D H (2007). J Am Chem Soc.

[29] Hornemann Y, Kehrer J P, Nunez C S, Ranieri R L (1974). J Am Chem Soc.

[30] Hornemann U, Corcoran J W (1981). Biosynthesis of the mitomycins. Biosynthesis..

[31] Varoglu M, Mao Y, Sherman D H (2001). J Am Chem Soc.

[32] Anderson G M, Kibby J J, Rickards R W, Rotschild J M (1980). J Chem Soc, Chem Commun.

[33] Kennedy K A, Rockwell S, Sartorelli A C (1980). Cancer Res.

[34] Sartorelli A C (1988). Cancer Res.

[35] Kumar G S, He Q Y, Behr-Ventura D, Tomasz M (1995). Biochemistry.

[36] Rodighiero G, Marciani Magno S, Dell Acqua F, Vedaldi D (1978). Farmaco Ed Sci.

[37] Penketh P G, Hodnick W F, Belcourt M F, Shyam K, Sherman D H, Sartorelli A C (2001). J Biol Chem.

[38] Cera C, Egbertson M, Teng S P, Crothers D M, Danishefsky S J (1989). Biochemistry.

[39] Keyes S R, Fracasso P M, Heimbrook D C, Rockwell S, Sligar S G, Sartorelli A C (1984). Cancer Res.

[40] Iyer V N, Szybalski W (1964). Science.

[41] Andrews P A, Pan S S, Bachur N R (1986). J Am Chem Soc.

[42] Kohn H, Zein N, Lin X Q, Ding J Q, Kadish K M (1987). J Am Chem Soc.

[43] Egbertson M, Danishefsky S J (1987). J Am Chem Soc.

[44] Danishefsky S J, Egbertson M J (1986). J Am Chem Soc.

[45] Moore H W (1977). Science.

[46] Kumar G S, Lipman R, Cummings J, Tomasz M (1997). Biochemistry.

[47] Prakash A S, Beall H, Ross D, Gibson N W (1993). Biochemistry.

[48] Danishefsky S, Ciufolini M (1984). J Am Chem Soc.

[49] Hoey B M, Butler J, Swallow A J (1988). Biochemistry.

[50] Kalyanaraman B, Perez-Reyes E, Mason R P (1980). Biochim Biophys Acta.

[51] Boger D L, Trost B M, Fleming I (1991). Heterodiene Additions. Comp. Org. Syn..

[52] McClure K F, Danishefsky S J (1993). J Am Chem Soc.

[53] Auerbach J, Franck R W (1969). Chem Commun.

[54] Franck R W, Bernady K F (1968). J Org Chem.

[55] Franck R W, Tomasz M, Wilman D E V (1989). Chemistry of Anti-Tumor Agents;.

[56] Trost B M, Pearson W H (1981). J Am Chem Soc.

[57] Naruta Y, Nagai N, Arita Y, Maruyama K (1987). J Org Chem.

[58] Naruta Y, Nagai N, Arita Y, Maruyama K (1988). J Chem Soc, Perkin Trans 1.

[59] Hudlicky T, Frazier J O, Seoane G, Tiedje M, Seoane A, Kwart L D, Beal C (1986). J Am Chem Soc.

[60] Weyler W, Pearse D S, Moore H W (1973). J Am Chem Soc.

[61] Caramella P, Rondan N G, Paddon-Row M N, Houk K N (1981). J Am Chem Soc.

[62] Judd T C, Williams R M (2002). Angew Chem, Int Ed.

[63] Ducept P, Gubler D A, Williams R M (2006). Heterocycles.

[64] Rollins S B, Williams R M (1997). Tetrahedron Lett.

[65] Dembinski R (2004). Eur J Org Chem.

[66] Namiki H, Chamberland S, Gubler D A, Williams R M (2007). Org Lett.

[67] Fuchs P L (1974). J Am Chem Soc.

[68] Steward J M, Westberg H H (1965). J Org Chem.

[69] Dolfini J E, Menich K, Corliss P, Danishefsky S J, Cavanaugh R, Chakrabartty S (1966). Tetrahedron Lett.

[70] Corey E J, Fuchs P L (1972). J Am Chem Soc.

[71] Bone T A, Perkin W H (1895). J Chem Soc.

[72] Danishefsky S J, Rovnyak G (1975). J Org Chem.

[73] Danishefsky S J, Singh R K (1975). J Org Chem.

[74] Danishefsky S J, Singh R K (1975). J Am Chem Soc.

[75] Danishefsky S J (1979). Acc Chem Res.

[76] Singh R K, Danishefsky S J, Freeman J P (1990). Organic Syntheses.

[77] Danishefsky S, Doehner R (1977). Tetrahedron Lett.

[78] Danishefsky S, Regan J, Doehner R (1981). J Org Chem.

[79] Danishefsky S, McKee R, Singh R K (1977). J Am Chem Soc.

[80] Sharpless K B, Lauer R F (1974). J Org Chem.

[81] Sharpless K B, Lauer R F (1973). J Am Chem Soc.

[82] Danishefsky S, Berman E M, Ciufolini M, Etheredge S J, Segmuller B E (1985). J Am Chem Soc.

[83] Nicolaou K C, Clarmon D A, Narnette W E, Sietz S P (1979). J Am Chem Soc.

[84] Nicolaou K C (1981). Tetrahedron.

[85] Kinoshita S, Uzu K, Nakano K, Shimizu M, Takahashi T, Matsui M (1971). J Med Chem.

[86] Danishefsky S J, Feigelson G B (1987). Heterocycles.

[87] Ziegler F E, Belema M (1997). J Org Chem.

[88] Grierson D (1990). Org React.

[89] Ziegler F E, Berlin M Y (1998). Tetrahedron Lett.

[90] Kende A S (1986). Organic Syntheses.

[91] Seebach D, Wasmuth D (1980). Helv Chim Acta.

[92] Mori K, Iwasawa H (1980). Tetrahedron.

[93] Dunigan J, Weigel L O (1991). J Org Chem.

[94] Ittah Y, Sasson Y, Shahak I, Tsaroom S, Blum J (1978). J Org Chem.

[95] Ziegler F E, Berlin M Y, Lee K, Looker A R (2001). Org Lett.

[96] Gardner J N, Carlon F E, Gnoj O (1968). J Org Chem.

[97] Vedejs E, Little J (2001). J Am Chem Soc.

[98] Yet L (2000). Chem Rev.

[99] Holemann A, Reissig H U (2004). Synlett.

[100] Dobbs A P, Jones K, Veal K T (1995). Tetrahedron Lett.

[101] Kozokowski A P, Mugrage B B (1988). J Chem Soc, Perkin Trans 1.

[102] Kambe M, Arai E, Suzuki M, Tokuyama H, Fukuyama T (2001). Org Lett.

[103] Reinhoudt D N, Visser G W, Verboom W, Benders P H, Pennings M L M (1983). J Am Chem Soc.

[104] Dijksman W C, Verboom W, Egberink J M, Reinhoudt D N (1985). J Org Chem.

[105] Verboom W, Lammerink B H M, Egberink R J M, Reinhoudt D N, Harkema S (1985). J Org Chem.

[106] Lee S, Lee W M, Sulikowski G A (1999). J Org Chem.

[107] Lim H J, Sulikowski G A (1996). Tetrahedron Lett.

[108] Baranano D, Mann G, Hartwig J F (1997). Curr Org Chem.

[109] Hartwig J F (1997). Synlett.

[110] Hartwig J F (1998). Angew Chem, Int Ed Engl.

[111] Martinez L E, Leighton J L, Carsten D H, Jacobsen E N (1995). J Am Chem Soc.

[112] Kuwajima I, Urabe H (1982). J Am Chem Soc.

[113] Wang P, Adams J (1994). J Am Chem Soc.

[114] Lee S, Lim H, Cha K L, Sulikowski G A (1997). Tetrahedron.

[115] Allan G M, Parson A F, Pons J F (2002). Synlett.

[116] Kulinkovich O G, Sviridov S V, Vasilevskii D A (1991). Synthesis.

[117] Lee J, Ha J D, Cha J K (1997). J Am Chem Soc.

[118] Finley K T, Patai S (1974). The addition and substitution chemistry of quinones. Chemistry of the Quinonoid Compounds.

[119] Watson I D, Yu L, Yudin A K (2006). Acc Chem Res.

[120] Balachari D, O’Doherty G A (2000). Org Lett.

[121] Fujioka H, Christ W J, Cha J K, Leder J, Kishi Y, Uemura D, Hirata Y (1982). J Am Chem Soc.

[122] Tidwell T T (1990). Organic reactions.

[123] Boruah R C, Skibo E B (1995). J Org Chem.

[124] Kametani T, Kigawa Y, Nemoto H, Ihara M, Fukumoto K (1980). J Chem Soc, Perkin Trans 1.

[125] Kametani T, Takahashi K, Ihara M, Fukumoto K (1978). Heterocycles.

[126] Kametani T, Takahashi K (1978). Heterocycles.

[127] Kametani T, Takahashi K, Ihara M, Fukumoto K (1978). Heterocycles.

[128] Kametani T, Osawa T, Ihara M (1979). Heterocycles.

[129] Taylor W G, Remers W A (1975). J Med Chem.

[130] Eschenmoser A, Wintner C E (1977). Science.

[131] Yamada Y, Miljkovic D, Wehrli P, Golding B, Loeliger P, Keese R, Mueller K, Eschenmoser A (1969). Angew Chem.

[132] Gotschi E, Hunkeler W, Wild H J, Fuhrer W, Gleason J, Eschenmoser A (1973). Angew Chem, Int Ed Engl.

[133] Boyland E, Manson D (1951). J Chem Soc.

[134] Luly J R, Rapoport H (1983). J Am Chem Soc.

[135] Remers W A, Roth R H, Weiss M J (1965). J Org Chem.

[136] Taylor W G, Leadbetter G, Fost D L, Remers W A (1977). J Med Chem.

[137] Leadbetter G, Fost D L, Ekwuribe N N, Remers W A (1974). J Org Chem.

[138] Poletto J F, Allen G R, Weiss M J (1968). J Med Chem.

[139] Yamamoto Y, Asao N (1993). Chem Rev.

[140] Bloch R (1998). Chem Rev.

[141] Kobayashi S, Ishitani H (1999). Chem Rev.

[142] Shibata I, Nose K, Sakamoto K, Yasuda M, Baba A (2004). J Org Chem.

[143] Remuson R (2007). Beilstein J Org Chem.

[144] Coleman R S, Felpin F X, Chen W (2004). J Org Chem.

[145] He F, Bo Y, Altom J D, Corey E J (1999). J Am Chem Soc.

[146] Feigelson G B, Danishefsky S J (1988). J Org Chem.

[147] Williams A L, Srinivasan J M, Johnston J N (2006). Org Lett.

[148] Brown D G, Velthuisen E J, Commerford J R, Brisbois R G, Hoye T R (1996). J Org Chem.

[149] Shaw K J, Luly J R, Rapoport H (1985). J Org Chem.

[150] Michael J P, Koning C B, Mudzunga T T, Petersen R L (2006). Synlett.

[151] Dong W, Jimenez L (1999). J Org Chem.

[152] Wang Z, Jimenez L S (1994). J Am Chem Soc.

[153] Cory R M, Ritchie B M (1983). J Chem Soc, Chem Commun.

[154] Kametani T, Takahashi K, Ihara M, Fukumoto K (1978). J Chem Soc, Perkin Trans 1.

[155] Kametani T, Takahashi K, Ihara M, Fukumoto K (1977). Heterocycles.

[156] Gribble G W, Lord D, Skotnicki J, Dietz S E, Eaton J T, Johnson J L (1974). J Am Chem Soc.

[157] Kornblum N, Jones W J, Anderson G J (1959). J Am Chem Soc.

[158] Henbest H B, Wrigley T I (1957). J Chem Soc.

[159] Corey E J, Ursprung J J (1956). J Am Chem Soc.

[160] Allen G R, Polletto J F, Weiss M J (1965). J Org Chem.

[161] Kametani T, Takahashi K, Ihara M, Fukumoto K (1976). J Chem Soc Perkin Trans 1.

[162] Jones G B, Guzel M, Mathews J E (2000). Tetrahedron Lett.

[163] Feigelson G B, Egbertson M, Danishefsky S J (1988). J Org Chem.

[164] Ockenden D W, Schofield K (1953). J Chem Soc.

[165] Veysoglu T, Mitscher L A (1981). Tetrahedron Lett.

[166] Fukuyama T, Yang L (1989). J Am Chem Soc.

[167] Harada T, Iwai H, Takatsuki H, Fujita K, Kubo M, Oku A (2001). Org Lett.

[168] Remy P, Langner M, Bolm C (2006). Org Lett.

[169] Bluet G, Campagne J M (2001). J Org Chem.

[170] Wadamoto M, Ozasa N, Yanagisawa A, Yamamoto H (2003). J Org Chem.

[171] Jankowska J, Mlynarski J (2006). J Org Chem.

[172] Carlsen P H J, Katsuki T, Martin V S, Sharpless K B (1981). J Org Chem.

[173] Ohnuma T, Sekine Y, Ban Y (1979). Tetrahedron Lett.

[174] Oda K, Ohnuma T, Ban Y (1984). J Org Chem.

[175] Nakajima S, Yoshida K, Mori M, Ban Y, Shibasaki M (1990). J Chem Soc, Chem Commun.

[176] Yoshida K, Nakajima S, Ohnuma T, Ban Y, Shibasaki M, Aoe K, Date T (1988). J Org Chem.

[177] Greene T W, Wuts P G (1999). Protective goups in organic synthesis.

[178] Oda K, Ohnuma T, Ban Y, Aoe K (1984). J Am Chem Soc.

[179] Wakamatsu T, Nishi T, Ohnuma T, Ban Y (1984). Synth Commun.

[180] Kubo I, Kim M, Ganjian I, Kamikawa T, Yamagiwa Y (1987). Tetrahedron.

[181] Hirama M, Iwashita M, Yamazaki Y, Ito S (1984). Tetrahedron Lett.

[182] Vedejs E, Klapars A, Naidu B N, Piotrowski D W, Tucci F C (2000). J Am Chem Soc.

[183] Vedejs E, Naidu B N, Klapars A, Warner D L, Li V S, Na Y, Kohn H (2003). J Am Chem Soc.

[184] Ito H, Taguchi T, Hanzawa Y J (1993). J Org Chem.

[185] Bobeck D R, Warner D L, Vedejs E (2007). J Org Chem.

[186] Warner D L, Hibberd A M, Kalman M, Klapars A, Vedejs E (2007). J Org Chem.

[187] Ducray R, Ciufolini M A (2002). Angew Chem, Int Ed.

[188] Ciufolini M A, Deaton M V, Zhu S, Chen M (1997). Tetrahedron.

[189] Ciufolini M A (2005). Il Pharmaco.

[190] Ducray R, Cramer N, Ciufolini M A (2001). Tetrahedron Lett.

[191] Reetz M T, Steinbach R, Westermann J, Peter R, Wenderoth B (1985). Chem Ber.

[192] Alonso F, Beletskaya I P, Yus M (2004). Chem Rev.

[193] Anand N K, Carreira E M (2001). J Am Chem Soc.

[194] Deiters A, Martin S F (2004). Chem Rev.

[195] Tsuboike K, Guerin D J, Mennen S M, Miller S J (2004). Tetrahedron.

[196] Davie E A C, Mennen S M, Xu Y, Miller S J (2007). Chem Rev.

[197] González-Pérez P, Pérez-Serrano L, Casarrubios L, Dominguez G, Pérez-Castells J (2002). Tetrahedron Lett.

[198] Chamberland S, Grüschow S, Sherman D H, Williams R M (2009). Org Lett.

[199] Van Lanen S G, Shen B (2006). Drug Discovery Today: Technologies.

[200] Mao Y, Varoglu M, Sherman D H (1999). Chem Biol.

